# Expected lifetime numbers and costs of fractures in postmenopausal women with and without osteoporosis in Germany: a discrete event simulation model

**DOI:** 10.1186/1472-6963-14-284

**Published:** 2014-06-30

**Authors:** Florian Bleibler, Kilian Rapp, Andrea Jaensch, Clemens Becker, Hans-Helmut König

**Affiliations:** 1 Department for Health Economics and Health Service Research, Hamburg Center for Health Economics, University Medical Center Hamburg-Eppendorf, Martinistr. 52, D-20246 Hamburg, Germany; 2 Department of Clinical Gerontology, Robert-Bosch-Hospital, Auerbachstr 110, D-70376 Stuttgart, Germany; 3 Institute of Epidemiology and Medical Biometry, Ulm University, Helmholtzstr 22, D-89081 Ulm, Germany

**Keywords:** Discrete event simulation, Fractures, Health care costs, Osteoporosis

## Abstract

**Background:**

Osteoporotic fractures cause a large health burden and substantial costs. This study estimated the expected fracture numbers and costs for the remaining lifetime of postmenopausal women in Germany.

**Methods:**

A discrete event simulation (DES) model which tracks changes in fracture risk due to osteoporosis, a previous fracture or institutionalization in a nursing home was developed. Expected lifetime fracture numbers and costs per capita were estimated for postmenopausal women (aged 50 and older) at average osteoporosis risk (AOR) and for those never suffering from osteoporosis. Direct and indirect costs were modeled. Deterministic univariate and probabilistic sensitivity analyses were conducted.

**Results:**

The expected fracture numbers over the remaining lifetime of a 50 year old woman with AOR for each fracture type (% attributable to osteoporosis) were: hip 0.282 (57.9%), wrist 0.229 (18.2%), clinical vertebral 0.206 (39.2%), humerus 0.147 (43.5%), pelvis 0.105 (47.5%), and other femur 0.033 (52.1%). Expected discounted fracture lifetime costs (excess cost attributable to osteoporosis) per 50 year old woman with AOR amounted to €4,479 (€1,995). Most costs were accrued in the hospital €1,743 (€751) and long-term care sectors €1,210 (€620). Univariate sensitivity analysis resulted in percentage changes between -48.4% (if fracture rates decreased by 2% per year) and +83.5% (if fracture rates increased by 2% per year) compared to base case excess costs. Costs for women with osteoporosis were about 3.3 times of those never getting osteoporosis (€7,463 vs. €2,247), and were markedly increased for women with a previous fracture.

**Conclusion:**

The results of this study indicate that osteoporosis causes a substantial share of fracture costs in postmenopausal women, which strongly increase with age and previous fractures.

## Background

With a lifetime risk of 40% to 50%, an osteoporotic fracture is one of the most likely negative health events in the remaining lifetime of a 50 year old woman [1]. The individual probability for a 50 year old woman of experiencing an osteoporotic fracture is higher than the probability of developing an atherosclerotic cardiovascular disease (4.1% to 30.7% [2] (depending on risk profile)) or breast cancer (around 11% [3]).

Studies have shown that a decrease in bone mineral density (BMD) is a strong predictor for fractures on different sites of the human body [4-6]. According to the World Health Organization (WHO) a person suffers from osteoporosis when the person’s BMD is at least 2.5 standard deviations lower (T≤-2.5) than the average BMD of healthy adults [7]. Clinically, osteoporosis is characterized ”by low bone mass and microarchitectural deterioration of bone tissue, leading to enhanced bone fragility and consequent increase in fracture risk” [8]. While women are more often affected than men, a strong increase in osteoporosis prevalence rates beyond the age of 50 occurs in both genders [9,10].

Osteoporotic fractures have serious consequences on the individual and the societal level. Fracture patients often have a decreased functional mobility [11], health related quality of life [12] and are faced with an increased mortality [13]. Especially for advanced elderly persons, fracture related functional impairment often necessitates external help from professional home care services, relatives or friends [11]. In some cases institutionalization in a nursing home (NH) is unavoidable [14]. On the societal level fractures have repeatedly been shown to cause a high economic burden [15-21]. As a consequence of the (expected) demographic change in Germany [22], the economic burden due to osteoporotic fractures is likely to increase strongly in the next decades [17].

Internationally, the economic burden of osteoporosis and osteoporotic fractures has frequently been investigated in the last decade [16,18-21,23]. In contrast to the large number of cost studies worldwide, the evidence for Germany is not abundant. Four of the available cost studies on osteoporotic fractures were prevalence based, estimating fracture costs for a specific year [24-27]. One study used an incidence based approach to estimate lifetime costs caused by hip fractures [28]. Another study used a Markov-cohort model to estimate the cumulative (2010–2050) and yearly (2010, 2030 and 2050) osteoporosis-attributable costs of different fracture types [17]. None of these studies estimated expected numbers and costs for all relevant fractures occurring over the remaining lifetime. Moreover, expected costs of specific subgroups, e.g. persons already suffering from osteoporosis, having a prevalent fracture or living in a NH, were not analyzed.

Our study had different aims. First, a discrete event simulation model (DES) was developed to estimate the expected lifetime fracture numbers and costs of six osteoporotic fracture types for 50 year old women in Germany. Second, the same estimates for hypothetical women who never suffer from osteoporosis over their remaining lifetime were made. Third, lifetime fracture costs attributable to the risk factor osteoporosis (excess cost) were calculated which indicate the economic potential of preventing osteoporosis as the main risk factor for fractures. Finally, lifetime fracture costs were estimated for women with different risk profiles (with previous fractures, with osteoporosis, or living in a NH) and at different starting ages. In summary, our model should give a deeper insight into the economics of six osteoporotic fractures in Germany, and should provide new and valuable information to the international literature.

## Methods

### Modeling approach

A DES model [29-31] was developed to estimate the expected lifetime fracture numbers and costs of postmenopausal women (aged 50 years and older) in the German general population (at average risk getting osteoporosis) and in those who never suffer from osteoporosis. The simulation starts in the year 2009 and simulates 50 year old women until the age of 100 years or death (lifetime horizon). The included costs are considered from a societal perspective.

A DES is an individual based simulation technique where all individuals are simulated one by one [30]. The main components in DES are entities, attributes, events, time, resources (optional) and queues (optional) [29]. In a disease simulation context entities are individuals (e.g. patients). Individuals have attributes which reflect different personal characteristics, for example age, gender, health status, risk factors or event history. Depending on the number and combinations of attributes the model determines the individual’s probability to experience an event. The individual’s attributes profile is dynamic and can change at each time point in the model. Changes can be triggered by time alone (e.g. due to natural aging) or by events (e.g. occurrence of osteoporosis). Events are dichotomous (discrete) and can occur simultaneously at each time point (interval) in the model [29]. Event initialization is done by comparing a uniform (pseudo) random number between 0 and 1 with an event probability or a value of a hazard function. An event occurs when the random number is smaller (or equal) than the event probability value [30]. Time can be handled in two different ways in DES: In an “event driven” simulation time jumps from event to event, whereas in a “time driven” simulation time progresses by constant intervals (e.g. years, months, days) [31]. Optionally, DES can also handle resource constraints using queuing-systems and allow for interaction between individuals [29].

**Figure 1:**
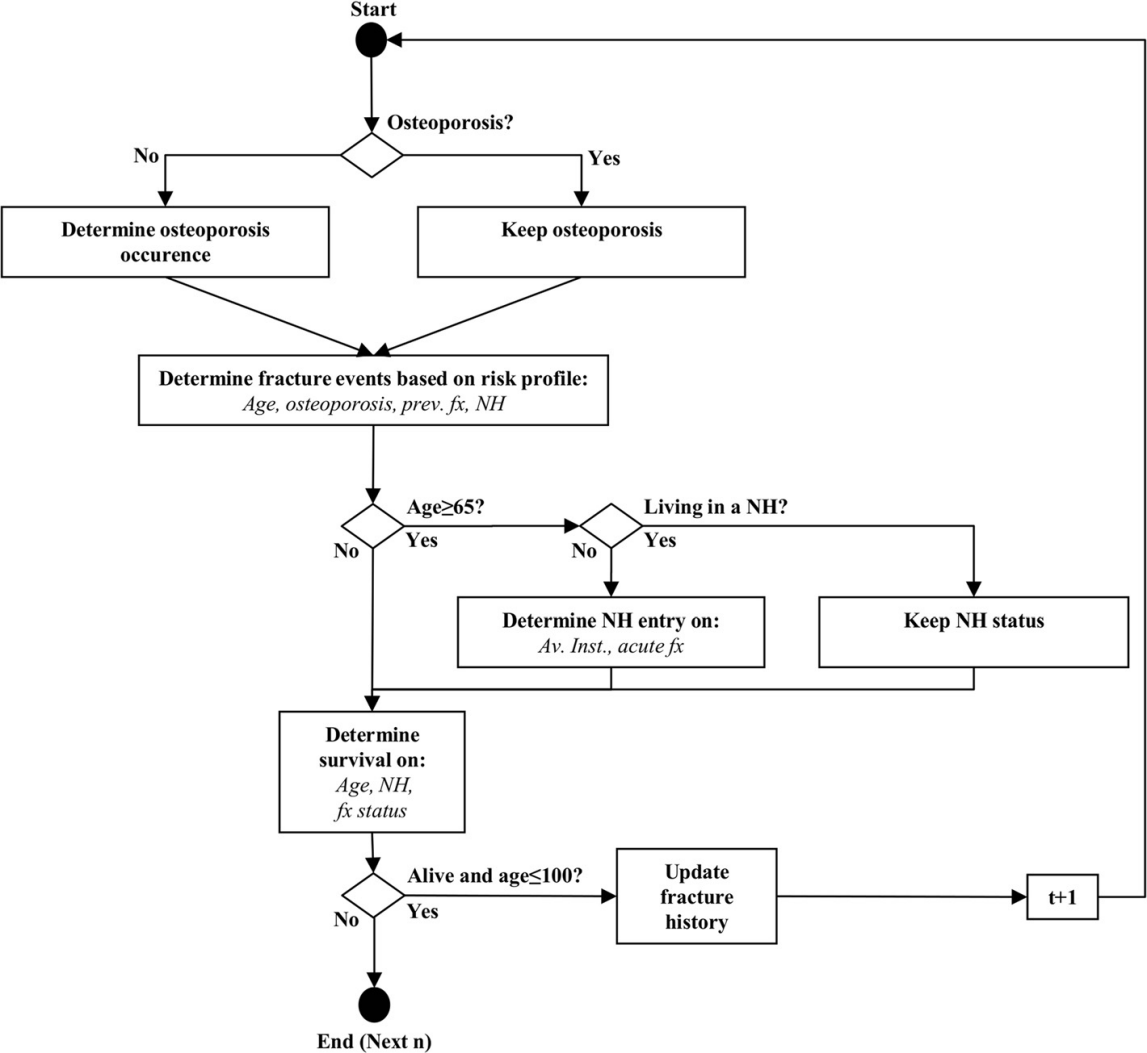
**Model process and structure (Flow Chart).** Legend of Figure 1: prev. fx = previous fracture, NH = nursing home, av. Inst. = average NH institutionalization probability, acute fx = acute fracture, fx status = acute or previous fracture, t = time interval, n = individual.

In order to estimate the expected lifetime fracture numbers and costs of a 50 year old woman, we conceptualized a “time driven” DES with time intervals of one year, no resource constraints and no interaction between individuals. The patient flow diagram in Figure 1 shows the mechanism of the DES-model: “Circles” represent start- and end-point of each simulation run (simulation of one individual n); in “rectangles” events are initialized whereas “rhombuses” represent decision points which determine the individual’s way through the model based on the individual’s attributes. The main events in this DES are the occurrences of hip, other femur, clinical vertebral, humerus, pelvis and wrist fractures. The probability of a fracture event is influenced by four risk factors (attributes) known from the literature: Age [32,33], osteoporosis (BMD) [4-6], prevalent fractures (prev. fx) [34], and living in a NH [35,36]. The occurrence of a risk factor itself is modeled as an event, e.g. institutionalization in a NH, and can arise in each time interval. At the beginning of each simulation run for an individual n the model determines if the individual directly starts with osteoporosis or a prevalent fracture (preload) [29], based on the age specific prevalence rates of these risk factors. All women start with the age of 50 in the base case.

There is evidence that fractures increase the probability of institutionalization in a NH [14]. Therefore, the probability of the event “admission to a NH” (NH entry) is dependent on acute fracture events (acute fx) and the age-dependent average institutionalization probability (av. Inst.). Fractures increase mortality in the year of the fracture and in the subsequent years [13]; furthermore it is known that individuals living in a NH have higher mortality rates compared to those who do not at the same age [37]. Therefore, whether an individual survives the actual time interval (“Survival”) depends on age, the presence of fractures (fx status) in the actual and previous time interval and the living situation (living in NH or not). Each individual is simulated until one of the two endpoint conditions, age of 100 years or death, is fulfilled. The simulation was programmed in Matlab R2012a (The MathWorks, Inc., Natick, MA, USA) in combination with Excel 2010 (Microsoft Cooperation, Redmond, WA, USA).

### Cost tracking

Not all fracture patients are treated in an inpatient setting (hospital). Therefore, the model determines on the basis of fracture specific hospitalization probabilities, whether a fracture patient is treated in a hospital or exclusively treated in an outpatient setting. If a patient is treated in a hospital, the model tracks acute hospital and post-hospital outpatient treatment costs; otherwise only outpatient costs are tracked. Also, not all patients receive inpatient rehabilitation after a hospital stay. For this reason, the same cost tracking approach, based on fracture specific post-hospital rehabilitation probabilities, was applied for rehabilitation costs. For tracking fracture-attributable long term care costs, a parallel background simulation approach was implemented [20]. The approach simulates the probability of institutionalization in a NH for any reason and the fracture specific probability in parallel. If a woman is institutionalized due to a fracture, long term care costs (i.e. yearly NH costs weighted by level of care as well as capital costs) are tracked as fracture-attributable only as long as no institutionalization occurs for any other reason [20]. A half-cycle correction was applied to long term care costs [38].

All other cost categories in the model are tracked as unit costs (rewards) per fracture event, similar to the common cohort model approach [38]. A detailed description of the epidemiological and cost specific input data used can be found in the electronic supplementary material (Additional file 1).

### Determination of life time costs and fracture events

In total, two risk groups of 200,000 hypothetical women (simulation runs) each were simulated through the model. For the first risk group it was assumed that these women were at average osteoporosis risk over their remaining lifetime (based on osteoporosis incidence and prevalence), whereas the second risk group was assumed to be free of osteoporosis over their remaining lifetime. To determine osteoporosis-attributable fracture costs (excess costs), the difference in costs between the two considered risk groups was calculated [17]. To eliminate the stochastic noise between the two groups (with regard to simulated costs and fracture numbers), that is the randomly generated difference between the two groups because of using different random streams (sequence of random numbers) for each group, the same random stream (common random numbers) was used for both simulated groups [39].

### Epidemiological input data

#### Fracture probabilities – general population

Age-dependent fracture probabilities for all considered fracture types were calculated based on official German population [22] and hospital discharge data [40] of the year 2009. The number of hospitalized fracture cases for women between 50 and 100 years of age (five-year age classes) were identified using the international classification of disease (ICD-10) with the following codes: hip S72.0-2, other femur S72.3-9, clinical vertebral S12.0-2/S12.7/S22.0-1/S32.0, humerus S42.2-4, pelvis S32.1-8 and wrist S52.5-6. Fracture probabilities based on hospital cases were estimated by dividing the identified fracture cases from the discharge statistic with the number of persons in the general population in the corresponding five-year age class (population at risk). While 100% of hip and other femoral fractures are treated in an inpatient setting (hospital) [41], some of clinical vertebral, humerus, pelvis and wrist fractures are treated exclusively in an outpatient setting [41-44]. Therefore, the estimated fracture probabilities calculated from hospital discharge statistics and population data underestimate the “total” fracture probabilities, at least for clinical vertebral, humerus, pelvis and wrist fractures. To include these outpatient fracture cases in the total fracture probability, the fracture probabilities estimated based on hospital cases were divided by fracture specific and age-independent hospitalization probabilities from the literature (clinical vertebral [41,44,45], humerus [42], pelvis [43], wrist [42]) (see Additional file 1: A.3.a-b).

The calculated “total” fracture probabilities pertain to the German female general population (see Table 1). However, as women were assumed to be in specific living situations (women with or without osteoporosis, women with or without a previous fracture, community-dwelling women or women living in a NH), these general population fracture probabilities had to be adjusted.

**Table 1:** Total yearly fracture probability of the female general population by age and fracture type

**Age**	**Hip**	**Other femur**	**Clinical vertebral**	**Humerus**	**Pelvis**	**Wrist**
50-54	0.00038	0.00010	0.00095	0.00085	0.00018	0.00221
55-59	0.00071	0.00014	0.00144	0.00143	0.00028	0.00390
60-64	0.00104	0.00021	0.00192	0.00194	0.00038	0.00491
65-69	0.00187	0.00033	0.00316	0.00272	0.00071	0.00620
70-74	0.00334	0.00054	0.00456	0.00360	0.00127	0.00684
75-79	0.00772	0.00102	0.00634	0.00530	0.00285	0.00866
80-84	0.01605	0.00162	0.01132	0.00716	0.00544	0.00973
85-89	0.02791	0.00262	0.01378	0.00872	0.00890	0.00916
90-94	0.03625	0.00324	0.01339	0.00861	0.01172	0.00740
95+	0.03960	0.00382	0.01052	0.00795	0.01118	0.00530

#### Fracture probabilities for women with and without a previous fracture

Clinical studies showed that a previous fracture is an important risk factor for a subsequent fracture, even after adjustment for BMD [34]. The age-adjusted risk ratios for further hip (RR_HipPreviousFx__vs.__NoPreviousFx_) and other osteoporotic fractures (RR_OsteoPreviousFx vs. NoPreviousFx_), comparing women with a previous fracture to those without a previous fracture, were taken from a meta-analysis [34]. However, the risk ratios reported in this meta-analysis are only applicable to fracture probabilities of women without a previous fracture. Therefore, in a first step, age-dependent relative risks for women without a previous fracture compared to the general population (RR_NoPreviousFx vs. GpFx_) were estimated. The calculation method was adopted from Schousboe et al. [46] using the following formula:

$$\begin{array}{lll} {\mathrm{RR}}_{\mathrm{NoPreviousFx}\;\mathrm{vs}.\;\mathrm{GpFx}} & = & 1/\left(1+\left({\mathrm{RR}}_{\mathrm{AnyPreviousFx}\;\mathrm{vs}\;\mathrm{NoPreviousFx}}‒1\right)\right)\\ & & \left(\;\;*{\mathrm{Prev}}_{\mathrm{PreviousFx}}\right) \end{array}$$

The age-adjusted risk ratios for any fracture comparing women with previous fractures to those without a previous fracture adjusted for BMD (RR_AnyPreviousFx vs. NoPreviousFx_) and age-dependent prevalence rates for previous fractures (Prev_PreviousFx_) were both taken from the previously mentioned meta-analysis [34]. In the second step the estimated RR_NoPreviousFx vs. GpFx_ were multiplied with the fracture probabilities of the general population to obtain fracture probabilities for women without a previous fracture. If a woman suffered from a fracture in the model, further fracture probabilities were calculated by combining fracture probabilities for women without a previous fracture with RR_HipPreviousFx vs. NoPreviousFx_ and RR_OsteoPreviousFx vs. NoPreviousFx_ depending on the fracture type (see Additional file 1: A.3.c).

#### Fracture probabilities for women with and without osteoporosis

Fracture probabilities for women with osteoporosis were derived by multiplying the age-dependent female general fracture probabilities with age and fracture specific relative risks for women with osteoporosis compared to the female general population (RR_OST_). The RR_OST_ were estimated based on a method described by Kanis et al. [10]. Necessary input data such as fracture specific relative risks by a decrease of one standard deviation in BMD measured at the femoral neck (RR_fx)_[4-6], the osteoporosis BMD threshold [10], and age-dependent population BMD values (reference values from NHANES III) [47] were taken from different international studies. To reflect lower fracture probabilities for women without osteoporosis compared to the female general population, probabilities were estimated based on a method described by Bleibler et al. [17] (see Additional file 1: A.3.d-e).

#### Fracture probabilities for women living and not living in a NH

There is evidence that women living in a NH have a higher fracture risk compared to community-dwelling women [35,36]. To include this in our model, relative fracture risks for women residing in a NH and for those who do not, each compared to the female general population, were calculated. Basis for the calculation was a dataset (claims data) from a large German mandatory sickness fund (Allgemeine Ortskrankenkasse Bayern (AOK Bavaria)). This dataset contains individual data about level of long-term care (e.g. begin of care, admission to NH), hospital cases by ICD-10 and date of death for persons aged 65 years and older insured between January 2004 and June 2009. Relative fracture risks were determined on pooled cumulative fracture incidence rates from 2004–2008. Incident hospital fracture cases by fracture type were identified using the ICD-10 codes mentioned above. A detailed description of the dataset can be found in the electronic supplementary material (see Additional file 1: A.3.f-g).

### Probability of developing osteoporosis

The age-dependent probability of developing osteoporosis was calculated based on osteoporosis prevalence rates originally estimated from the NHANES III study [9,48]. The osteoporosis prevalence rate was transformed into a applicable transition probability (incidence) using a method described by Podgor and Leske [49] (see Additional file 1: A.2).

### Probability of institutionalization in a NH

Especially hip fracture patients in higher age groups often have persistent reductions in their functional abilities and mobility after a fracture event [11]. Moreover, they frequently depend on external care or need to be institutionalized as a result of a fracture [11]. Similar to hip fractures, other types of osteoporotic fractures have been shown to increase the probability of institutionalization [14]. To model the association of a fracture event and institutionalization, crude age and fracture specific probabilities of admission to a NH (within 3 months after hospitalization) were estimated based on claims data from the AOK Bavaria. Besides a fracture related transition into a NH, the model allows institutionalization due to other reasons. To include this, official long-term care prevalence rates from 2009 [50] were transformed into NH institutionalization probabilities [49]. A transition between the status community-dwelling and status NH was possible for women 65 years and older. Similar to other simulation studies, it was assumed that persons in a NH will remain there for the rest of their lives [51] (see Additional file 1: A.4.a-b).

### Mortality

As main source of mortality, an official generation life table (all-cause mortality) of women born in 1959 was used to reflect a realistic remaining life expectancy for a 50 year old woman living in the year 2009 (start year of the model) [52]. Depending on the actual model state (community dwelling, NH or fracture), age specific all-cause mortality was multiplied with state specific relative risks of mortality. Relative risks of mortality due to fractures were taken form a Canadian study [13]. The authors of this study determined the increase in relative mortality risk due to hip, clinical vertebral, proximal humerus, wrist and other fractures for five age classes and a follow up period of 10 years separated in 3 categories (1; 2–5; 6–10 years). They adjusted the calculated relative risks for co-morbidities and location of residence. Relative mortality risk for persons living in a NH or in a community-dwelling setting compared to the female general population were calculated based on the AOK Bavaria claims data (see Additional file 1: A.1.a-d).

### Cost input data

Fracture related unit costs were estimated from a societal perspective for Germany. The base year of cost evaluation was 2009 and a discount rate of 3% was applied [53]. Included fracture related direct cost were separated in *three main categories*: *inpatient costs* include costs due to acute hospital care, rehabilitation after hospitalization and long term care, *outpatient costs* include costs due to physician and physiotherapist visits, analgesics (medication) as well as home care, and *family costs* include informal care costs. An overview over the main direct unit costs is shown in Table 2. Productivity costs were included in the model and estimated based on the human capital [54] and friction cost approach [55].

**Table 2:** Overview of direct unit costs in € by cost category and fracture type

Cost category*	**Hip**	**Other femur**	**Clinical vertebral**	**Humerus**	**Pelvis**	**Wrist**
Hospital treatment (plus outpatient aftercare costs)	8,554	8,395	6,324	5,764	5,005	3,794
Rehabilitation (if required after hospitalization)	2,187	2,187	2,092	2,337	2,177	2,337
Outpatient costs (if no hospitalization required)	n. a.	n. a.	1,614	835	963	835
Professional home care (age > 65, not in NH)	2,174	2,174	2,212	937	2,174	525
Informal home care (age > 65, not in NH)	2,361	2,361	2,016	2,961	2,361	581
Yearly long term care cost (age > 65, in NH)	25,759	25,759	25,759	25,759	25,759	25,759

*A detailed description of all references and calculations can be found in the electronic supplementary material (see Additional file 1: B.1.a-f).

### Inpatient costs

In Germany, hospitals are reimbursed according to a dual system: Operating costs due to direct resource uses are paid on the basis of a German version of diagnosis related groups (G-DRG) by health insurance funds (private or mandatory), whereas capital costs are compensated by federal states [53]. Therefore, applied hospital costs consist of fracture related DRGs and capital costs. To determine the DRG part of total hospital costs per fracture type, the G-DRG Browser V2010 [56] was used. This dataset includes information on a large representative sample of German hospitals in 2009, including number of DRG-cases, age, gender, disease diagnosis (ICD-10), DRG relative cost weights and mean length of stay. On the basis of this information an average relative cost weight and mean length of stay was calculated for each considered fracture type and multiplied with a state weighted base rate of 2009 [57] to obtain DRG costs. Capital costs were calculated by combining the fracture specific length of stay with a daily capital cost rate [58] (inflated to 2009 [59]) (see Additional file 1: B.1.a). *Rehabilitation* costs after a hospital stay were estimated by combining the fracture type related duration of an inpatient rehabilitation treatment [60] with a daily cost rate [61] (see Additional file 1: B.1.b). *Long term care* costs are based on the official care statistic 2009 [50]. This statistic provides information on daily care costs and number of persons living in long term care by level of care in 2009. Therefore, level of care weighted yearly inpatient long term care costs were calculated. Additionally, capital costs for long term care [62] were added to the yearly unit costs (see Additional file 1: B.1.c).

### Outpatient costs

Fracture related costs for physician visits (including outpatient surgeons), physiotherapeutic treatments and analgesics for patients exclusively treated in the outpatient sector, as well as outpatient cost for post-hospital treatment were estimated using German unit costs [58], inflated to 2009 [59]. Necessary resource use data in the three outpatient categories were taken from a German cost-effectiveness study [63]. The authors of this study collected information on resource use associated with hip, vertebral and wrist fractures. It was assumed that outpatient costs for other femur as well as pelvis fractures are similar to hip fractures and outpatient costs for humerus fractures are similar to wrist fractures (see Additional file 1: B.1.d).

Costs for home care per fracture type were determined based on information about fracture type specific hours of home care needed [19] and hourly unit costs [64]. As no information on fracture type specific hours of home care was available for Germany, a study from Austria was used [19]. It was assumed that only women older than 65 years utilize home care. No home care costs were applied to women living in a NH (see Additional file 1: B.1.e).

### Informal care costs

To reflect the fracture related costs of informal care [65], fracture type specific amount of hours spend by relatives of a fracture patient were evaluated with the market cost approach (proxy good) [65]. Each hour of care was monetarily valued by the hourly gross salary of an employee in the field of care for elderly and disabled persons [66], corrected by the employer share of social contribution [54,67]. As no information about number of hours spend by relatives of the fracture patients was available for Germany, Austrian data [19] were used. Similar to home care costs, it was assumed that women aged 65 or younger and women living in NH receive no informal care (see Additional file 1: B.1.f).

### Productivity costs

The model considers two categories of productivity costs. First, loss in productivity due to the fracture related inability to perform paid work, and second, the productivity loss due to osteoporosis related premature deaths [68]. Productivity loss was evaluated using the human capital [54] and the friction cost approach [55]. Necessary information, such as employment rate [69], yearly average gross earnings [66] corrected by employer share of social contribution [67], time away from work (due to disability and rehabilitation [60]), length of friction period [70] for the year 2009, were taken from official statistics. A yearly net wage increase of 2% was assumed [54,71]. If necessary, a half-cycle correction was applied to determine costs [38] (see Additional file 1: B.2.a-b).

### Model assumptions

In order to improve model transparency, all model assumptions are presented in Table 3, distinguishing between assumptions regarding event probabilities and costs. Model assumptions were evaluated subjectively with respect to their expected impact on modeling results and scored from 1 asterisk (*, low impact) to 3 asterisks (***, high impact).

**Table 3:** Overview of the model assumptions

**Assumptions regarding event probabilities**	**Impact**^**§**^
We applied osteoporosis prevalence rates and BMD-values from US-NHANES III reference data	***
We estimated “total” fracture probabilities by dividing fracture probabilities based on hospital cases with age-independent hospital probabilities	**
We assumed highest fracture related NH probability when more than one fractures occurs in the same time interval	**
We modeled fracture related entry in a NH only after a hospital stay	**
We assumed that only NH entries within 3 months after a fracture may be attributable to the fracture event itself	**
We applied age-dependent relative fracture risk by one standard deviation decrease in BMD to hip fractures and age-independent relative risks to other fractures	**
We assumed that osteoporosis risk attributions were calculated exclusively on BMD values measured at the femoral neck	**
We assumed that osteoporosis prevalence rates do not differ between women living in a NH and women who do not	*
We applied relative fracture risk and prevalence for previous fractures from an international meta-analysis	*
We applied relative fracture risk by one standard deviation decrease in BMD from international studies	*
We applied fracture mortality data from a Canadian study	*
We assumed the highest fracture excess mortality when more than one fracture occurs in the same time interval	*
We allowed first entry in a NH firstly for women aged 65 or older	*
We assumed that individuals in a NH remain there for their remaining lifetime	*
We assumed that patients with osteoporosis will have osteoporosis for their remaining lifetime	*
We allowed a maximum possible age of 100 years	*
**Assumptions regarding costing**	
We assumed that rehabilitation probabilities after a hospital stay do not differ between women living in NH and those who do not.	**
We applied Austria data for average hours of informal and professional home care by fracture type, also we assumed that the consumed hours are equivalent for hip, other femur and pelvis	**
We assumed age-dependent fracture unit costs	*
We assumed that the outpatient costs for humerus and wrist as well as the costs for pelvis, other femur and hip fractures are equivalent	*
We took outpatient resource use data from a study considering fracture patients with inflammatory bowel disease	*
We assumed that average informal and professional home care costs are only applicable for individuals not living in NH aged older than 65 years	*

^§^Expected impact on modeling results: * = low impact, ** = medium impact, *** = high impact.

### Scenario analysis

A number of scenarios of direct lifetime fracture costs were analyzed for women with different start characteristics. Each considered scenario consists of a combination of four of the following (model) start characteristics: Age (50 or 75), residential status (community dwelling (C) or NH (N)), disease status (already suffering from osteoporosis (O), at average risk of getting osteoporosis (avO) or not at risk of getting osteoporosis (nO)) and fracture history (with a previous fracture (P) or with no previous fracture (nP)). In total we modeled 18 scenarios, and each scenario was labeled based on the combination of the four start characteristics e.g. a 50 year old woman, community dwelling with average risk of getting osteoporosis and no previous fracture was labeled as 50_C_avO_nP in the results section. All scenarios were simulated separately, direct costs are presented undiscounted and discounted by 3%.

### Sensitivity analysis

Deterministic one way and probabilistic sensitivity analyses were conducted. We performed 20 deterministic sensitivity analyses (S1-S20) to evaluate the impact on excess costs due to osteoporosis-attributable fractures:

S1: We valued informal care based on the opportunity cost approach [65] with an average German 2009 hourly wage rate (corrected by employer share) [66,67]. S2: We valued informal care with a minimum hourly wage rate for employees in the area of health care and nursing introduced in 2010 in Germany [72]. S3: We assumed no excess mortality due to fractures. S4: We assumed baseline excess mortality only in the first year but not thereafter. S5: We assumed 30% of baseline excess mortality only in the first year, but none thereafter. S6: We did not consider a previous fracture as a risk factor for further fractures. S7: We applied the lower relative risk value of the 95% confidence interval to model the risk factor “previous fracture”. S8: We applied the upper relative risk value of the 95% confidence interval to model the risk factor “previous fracture”. S9: We applied the lower value for the fracture specific relative risks by a decrease of one standard deviation in BMD measured at the femoral neck. S10: We applied the upper value for the fracture specific relative risks by a decrease of one standard deviation in BMD measured at the femoral neck. S11: We increased the osteoporosis prevalence rate by +30%. S12: We decreased the osteoporosis prevalence rate by -30%. S13: We increased all input fracture probabilities by +30%. S14: We decreased all input fracture probabilities by -30%. S15: We assumed that only hip fracture patients have an increased risk to be institutionalized in a NH. S16: We assumed a yearly increase of modeled input fracture incidence rates by +1%. S17: We assumed a yearly decrease of modeled input fracture incidence rates by -1%. S18: We assumed a yearly increase of modeled input fracture incidence rates by +2%. S19: We assumed a yearly decrease of modeled input fracture incidence rates by -2%. S20: We applied a discount rate of 5%.

In order to perform a probabilistic sensitivity analysis, we simulated 10,000 hypothetical women for each risk group (simulation runs (n)) 1,500 times (simulations (m)) by varying parameters simultaneously for each of the 1,500 simulations (second-order uncertainty) [73]. Common distributional assumptions were used for costs, probabilities and relative risks [73] (see Additional file 1: C). The results of the probabilistic sensitivity analyses are reported as uncertainty intervals, estimated based on the percentile method (2.5 and 97.5 percentile) [73].

## Results

### Base case

**Figure 2:**
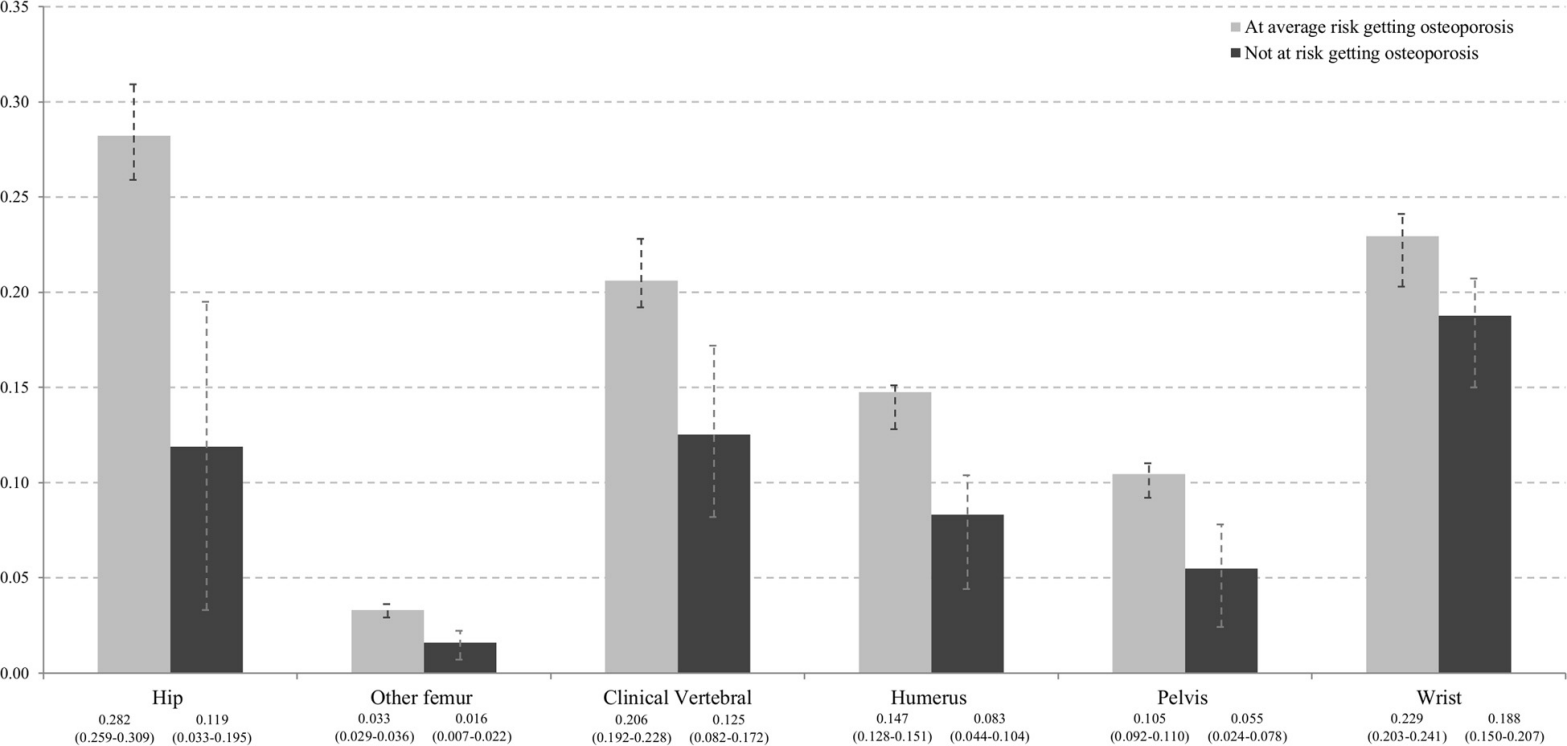
Expected lifetime numbers of fractures (95%UI) of a 50 year old woman by fracture type and risk class.

Figure 2 shows the expected lifetime numbers of the six analyzed fracture types of a 50 year old woman for the two risk groups. Considering a 50 year old woman with an average risk of getting osteoporosis, there are 0.282 hip fractures expected for her remaining life, whereas 0.119 hip fractures are expected for a woman never suffering from osteoporosis. Thus, around 57.9% of all expected hip fractures are attributable to osteoporosis. Wrist fractures are the second most frequent fracture type, with expected 0.229 fractures for women at average risk of osteoporosis and 0.188 for those not at risk. With around 18.2% of wrist fractures attributable to osteoporosis, this fracture type had the lowest osteoporosis risk attribution. The lifetime numbers of other femur, clinical verterbral, humerus and pelvis fractures for a woman with average osteoporosis risk are expected at 0.033, 0.206, 0.147 and 0.105 respectively, which corresponds to an osteoporosis risk attribution of around 52.1%, 39.2%, 43.5% and 47.5% for these fracture types.

Table 4 displays the expected direct costs for each of the six fracture types accrued in all considered healthcare sectors, excluding costs due to long term care. In women with average osteoporosis risk, hip fractures cause €1,277 (39.1%) of all discounted costs. Considering discounted excess cost due to osteoporosis, hip fractures cause €692 (50.4%) of total excess costs. The fracture types with the second and third largest contribution to discounted excess costs are clinical vertebral (15.9%) and humerus (14.9%) fractures. The lowest share of discounted excess costs was found for wrist fractures with 4.4%.

**Table 4:** Undiscounted and discounted fracture lifetime costs (€) of a 50 year old woman, by fracture type for two risk classes of osteoporosis and excess

***Direct cost***	**Undiscounted**						**Discounted (3%)**					
***By fracture type***	**Average risk (95%UI)**	**%**	**Not at risk (95%UI)**	**%**	**Excess (95%UI)**	**%**	**Average risk (95%UI)**	**%**	**Not at risk (95%UI)**	**%**	**Excess (95%UI)**	**%**
**Hip**	3,399	42.7	1,449	34.4	1,950	52.1	1,277	39.1	585	30.9	692	50.4
	(2,933-4,116)		(398–2,466)		(1,070-3,179)		(1,102-1,541)		(204–939)		(394–1,108)	
**Other femur**	388	4.9	188	4.5	200	5.3	157	4.8	85	4.5	72	5.2
	(316–461)		(83–270)		(106–331)		(126–181)		(44–114)		(39–115)	
**Clinical vertebral**	1,452	18.2	880	20.9	573	15.3	623	19.1	404	21.3	219	15.9
	(1,156-2,042)		(504–1,425)		(279–983)		(494–886)		(251–631)		(109–367)	
**Humerus**	1,182	14.9	664	15.8	518	13.8	532	16.3	328	17.3	204	14.9
	(951–1,377)		(320–846)		(299–829)		(422–611)		(180–395)		(121–318)	
**Pelvis**	773	9.7	413	9.8	360	9.6	295	9.0	169	8.9	126	9.2
	(617–958)		(173–646)		(176–609)		(236–364)		(83–253)		(63–209)	
**Wrist**	763	9.6	621	14.7	143	3.8	385	11.8	324	17.1	61	4.4
	(557–1,070)		(423–876)		(71–252)		(282–550)		(229–467)		(31–103)	
**Total**	7,958	100	4,214	100	3,744	100	3,269	100	1,895	100	1,374	100
	(6,883-8,940)		(2,027-6,018)		(2,045-5,939)		(2,814-3,664)		(1,064-2,538)		(774–2,134)	

95%UI = 95% uncertainty intervals; costs due to long term care are not included.

**Figure 3:**
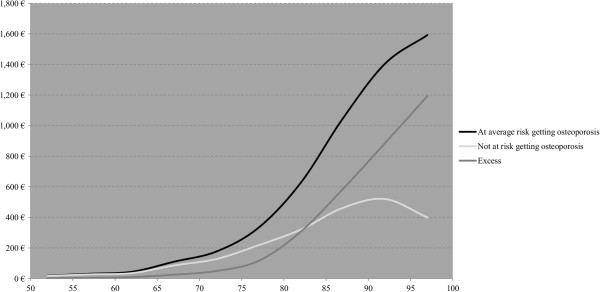
Annual fracture costs per capita by age for two risk classes and excess.

Table 5 displays the expected direct lifetime fracture costs of a 50 year old woman by healthcare sector. Considering all six fracture types, discounted total costs of a 50 year old woman at average risk getting osteoporosis amounted to €4,479, of which around €1,995 (44.5%) were excess costs. With regard to the different healthcare sectors, we found that around 71.3% of the discounted total excess costs resulted from inpatient treatment, mainly caused by acute hospital treatments (37.6%) and fracture-attributable long term care (31.1%). Treatments in the outpatient sector were responsible for 17.2% and informal care for 11.5% of these costs.Figure 3 depicts the expected annual undiscounted total direct fracture costs per capita for postmenopausal women by age. It shows that women with average osteoporosis risk aged 50–70 years have relatively low annual costs per capita of €14 to €142, with low osteoporosis cost attributions of 9.7% to 25.5%. However, a strong increase in these costs and corresponding cost attributions can be observed beyond the age of 75. The annual costs per capita increase from €260 for women aged 75 years to around €1,535 for those aged 95, with osteoporosis cost attributions of 31.3% to 68.0%. For women aged 82 and older, excess costs exceed costs for women never suffering from osteoporosis.

**Table 5:** Undiscounted and discounted fracture lifetime costs (€) of a 50 year old woman, by healthcare sector for two risk classes of osteoporosis and excess

***Direct cost***	**Undiscounted**						**Discounted (3%)**					
***By healthcare sector***	**Average risk (95%UI)**	**%**	**Not at risk (95%UI)**	**%**	**Excess (95%UI)**	**%**	**Average risk (95%UI)**	**%**	**Not at risk (95%UI)**	**%**	**Excess (95%UI)**	**%**
**Inpatient**												
Hospital	4,237	36.2	2,170	36.3	2,067	36.2	1,743	38.9	992	39.9	751	37.6
	(3,853-4,500)		(1,029-3,085)		(1,147-3,322)		(1,582-1,830)		(565–1,306)		(432–1,181)	
Rehabilitation	272	2.3	125	2.1	146	2.6	105	2.4	53	2.1	52	2.6
	(245–290)		(42–182)		(87–241)		(93–110)		(23–72)		(31–83)	
Long term care	3,731	31.9	1,758	29.4	1,973	34.5	1,210	27.0	590	23.7	620	31.1
	(3,349-4,159)		(475–2,834)		(920–3,481)		(1,071-1,324)		(185–913)		(285–1,073)	
**Outpatient**												
Physiotherapy, physician, analgesics	1,000	8.6	579	9.7	422	7.4	433	9.7	276	11.1	157	7.9
	(418–1,949)		(182–1,182)		(132–1,017)		(182–825)		(99–544)		(52–370)	
Prof. home care	1,105	9.5	605	10.1	500	8.7	443	9.9	257	10.4	186	9.3
	(800–1,401)		(275–925)		(262–843)		(320–561)		(130–379)		(100–308)	
**Family**												
Informal care	1,344	11.5	735	12.3	609	10.7	544	12.1	316	12.7	228	11.5
	(1,205-1,414)		(370–997)		(346–991)		(487–569)		(178–409)		(133–365)	
**Total**	11,689	100	5,973	100	5,716	100	4,479	100	2,485	100	1,995	100
	(10,406-12,929)		(2,533-8,757)		(2,988-9,466)		(3,942-4,898)		(1,258-3,424)		(1,076-3,215)	

*95*%*UI* = *95*% *uncertainty intervals*.

Considering discounted productivity costs due to the inability to perform paid work, we found only low excess lifetime costs per capita of around €63 applying the human capital approach and €22 applying the friction cost approach.

### Scenario analysis

**Figure 4:**
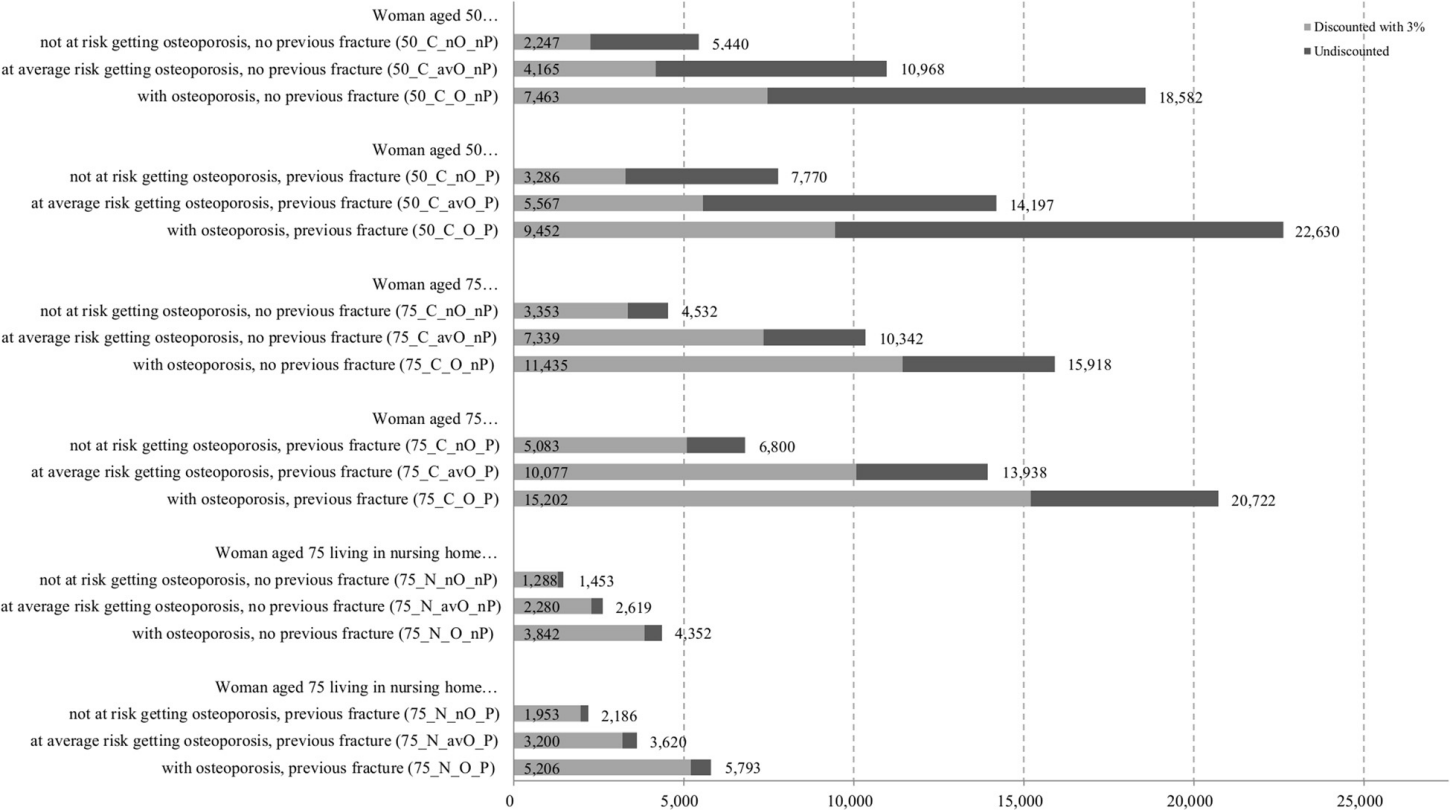
Undiscounted and discounted direct fracture lifetime costs (€) of different scenarios (women with different start characteristics).

The scenario analysis in Figure 4 shows that a 50 year old community-dwelling woman with osteoporosis and no previous fracture (50_C_O_nP) causes approximately 3.3 fold (€7,463 vs. €2,247) the discounted direct fractures lifetime costs of a 50 year old community-dwelling woman not at risk getting osteoporosis without a previous fracture (50_C_nO_nP). If a 50 year old community-dwelling woman with osteoporosis has additionally experienced a previous fracture (50_C_O_P), the fracture costs are 4.2 times (€9,452 vs. €2,247) higher. If a 50 year old community-dwelling woman never suffering from osteoporosis had a previous fracture (50_C_nO_P), discounted direct lifetime costs are increased by 46% compared to the same woman with no previous fracture (50_C_nO_nP). In general, we found that the expected discounted direct lifetime costs of a woman living in a NH are markedly lower than those of a woman living in a community dwelling setting. This is mainly explained by the high mortality in women institutionalized in a NH. Moreover, no costs for facture attributable long term care, professional home care and informal care were applied to institutionalized women.

### Sensitivity analysis

**Figure 5:**
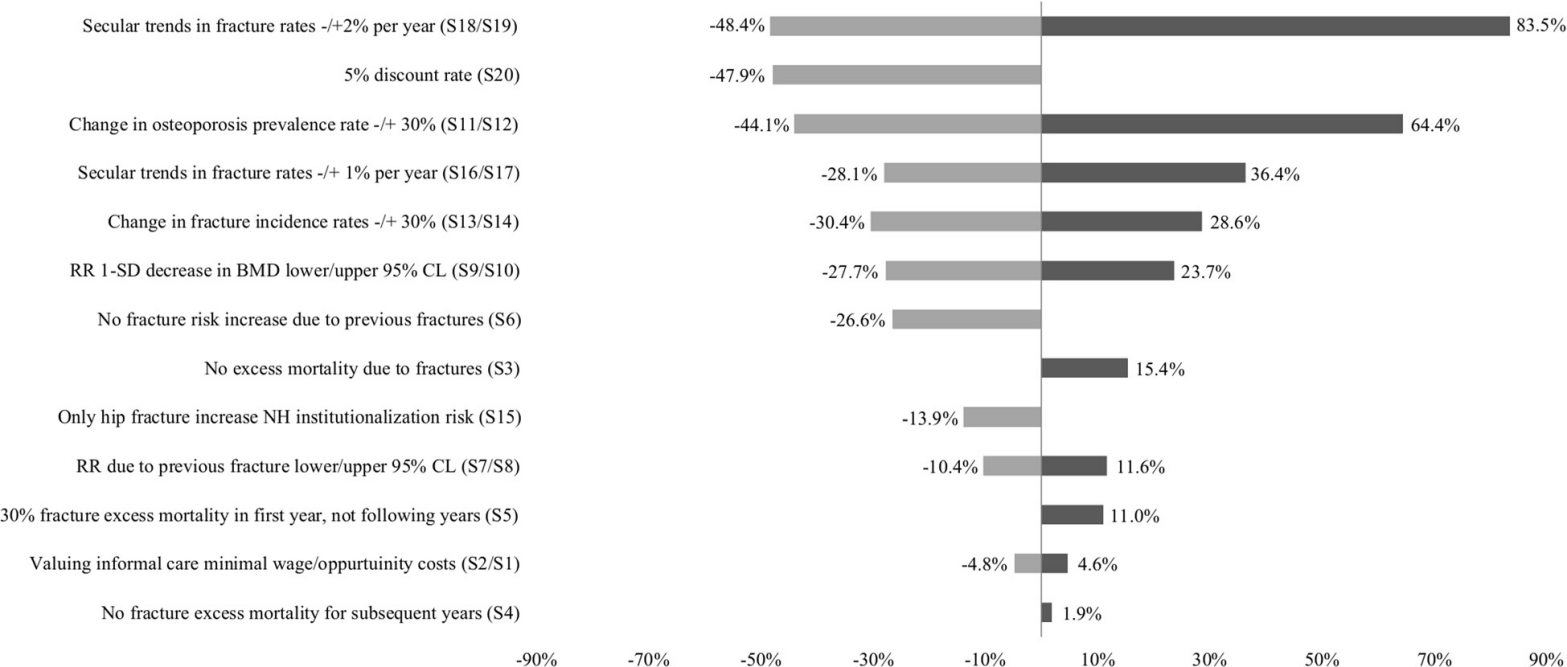
**Deterministic sensitivity analyses, difference (%) between direct fracture excess costs due to osteoporosis from base case excess costs.** Legend of Figure 5: CL = confidence limit; RR = relative risk; SD = standard deviation; BMD = bone mineral density.

The results of the deterministic sensitivity analyses are presented in Figure 5, whereas the results of the probabilistic sensitivity analysis (PSA) can be found in Tables 4, 5 and Figure 2 reported as 95% uncertainty intervals. Figure 5 shows the percentage difference (-%/+%) between each univariate sensitivity analysis and the discounted total direct fracture excess costs from the base case analysis. The largest percentage difference between costs was found when considering a yearly increase and decrease of fracture rates by -/+2% (S18/S19) (-48.4%/+83.5%), varying osteoporosis prevalence by -/+30% (S11/S12) (-44.1%/+64.4%), and by increasing the discount factor to 5% (S20) (-47.9%). The sensitivity analysis shows that neglecting the risk factor “previous fractures” (S6) reduces costs by 26.6%. Assuming that only hip fractures induce a fracture related institutionalization (S15) decreased costs by -13.9%. Applying minimal wage rates to value informal care (S2) led to a cost decrease of -4.8%, whereas applying the opportunity cost approach (S1) increased costs by +4.6%. Moreover, assuming no fracture excess mortality (S3) increased costs by 15.4%, while ignoring fracture related mortality effects beyond the year of fracture occurrence (S4) lead to a cost increase of 1.9%.

### Validation

For an *internal* validation of our model we compared the modeled fracture incidence rates with the expected fractures rates (input data) of the female general population. We also reported the distribution of the number of fractures per woman to support internal validity and transparency of the model estimates. Table 6 shows the expected and modeled hip fracture rates as well as the proportion of women with 0 to 6 hip fractures (the same data for all other fracture types is available in the electronic supplementary material (see Additional file 1: D.1.a-b)).

**Table 6:** Modeled and expected hip fracture rates and proportions of women with 0 to 6 hip fractures

**Age**	**Fracture rates**		**Fractures per woman**	**Proportion**
	expected	modeled	0	77.63%
50-54	0.0004	0.0003	1	17.57%
60-64	0.0010	0.0011	2	3.92%
70-74	0.0033	0.0034	3	0.75%
80-84	0.0161	0.0156	4	0.12%
90-95	0.0363	0.0355	5	0.02%
95+	0.0396	0.0400	6	0.00%

The expected and modeled fracture incidence rates are very similar (Table 6), which suggests that the proportions of women with different fracture probabilities (e.g. with and without a previous fracture, with and without osteoporosis, living in NH or not) are modeled appropriately.

**Figure 6:**
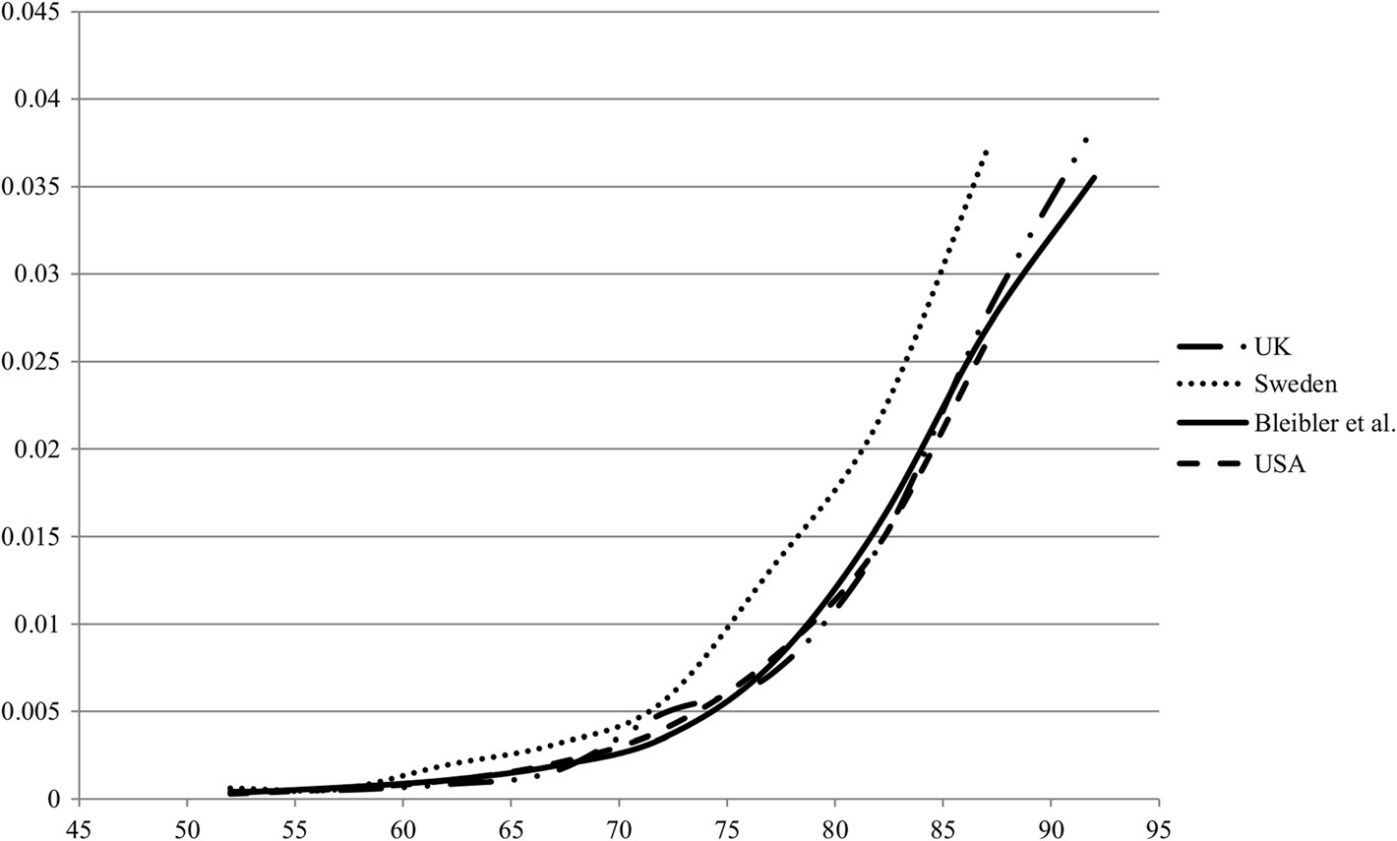
Comparison of modeled hip fracture rates with hip fracture rates from Sweden, UK, and USA.

As an *external* model validation, the modeled hip fracture incidence rates were compared to epidemiological studies from Sweden, USA and UK (Figure 6). Additionally, the fracture lifetime risk of a 50 year old woman at average risk of getting osteoporosis was compared to other modeling and epidemiological studies. Table 7 shows the (first ever) hip fracture lifetime risk of our modeling study in comparison with other epidemiological and modeling studies from different countries. Our estimated (first ever) fracture lifetime risk of 19.8% for hip fracture is in the range of other simulated lifetime hip fracture risk estimates. Furthermore, in comparison with epidemiological studies our estimated hip fracture lifetime risk and fracture rates (Table 7 and Figure 6) show a good fit. From a systematic review, comparing worldwide hip fracture risks, it is known that the hip fracture risk level in Germany should be between that of Sweden and USA [74], which is confirmed by our estimates. (The same data for all other fracture types is available in the electronic supplementary material (see Additional file 1: D.1.a-b)).

**Table 7:** Hip fracture lifetime risk from modeling and epidemiological studies

**Country**	**Study type**	**Hip**
Germany*	Model – present study	19.8%
Belgium** [75]	Model	29.0%
Australia* [76]	Model	17.0%
Switzerland [20]	Model	20.9%
USA*** [77]	Model	24.0%
Sweden* [33]	Epidemiological	22.9%
USA* [78]	Epidemiological	17.5%

*First ever lifetime risk of a 50 year old woman, **absolute fracture lifetime risk of 50 year old woman, ***First ever lifetime risk of a 65 year old woman.

## Discussion

### Summary and comparison to the literature

The goal of this study was to estimate the expected lifetime fracture numbers and costs per capita for two risk groups of German postmenopausal women, that is, women with average risk getting osteoporosis and women never suffering from osteoporosis. As key results we found that discounted lifetime fracture costs of €4,479 can be expected for a 50 year old woman with average osteoporosis and previous fracture risk, whereof €1,995 or 44.5% are attributable to the risk factor osteoporosis (excess costs). For both risk groups, around 70% of costs were due to inpatient treatment, like acute hospital treatments and long term care. The fracture type with the largest proportion of direct lifetime costs were hip fractures.

In a previous study [17], we estimated the impact of demographic change on osteoporosis-attributable fractures costs in Germany from 2010–2050. Since aggregated costs were calculated, a direct comparison in terms of lifetime costs per capita was not possible. The cost distribution between fracture types and healthcare sectors are quite similar in both studies, though. In contrast, in the present study it was possible to implement the event “NH entry” directly in the model, which allowed a more precise estimate of fracture attributable long term care costs. Internationally, few studies estimated and reported the average lifetime costs per capita of osteoporotic fractures. A study from Switzerland [20] found discounted (3%) inpatient lifetime costs for a person aged 50 (both genders) of CHF5,400 in the year 2000. In 2009 US$ purchasing power parities (US$PPP) [79] this equals US$PPP4,007, whereas we found average lifetime costs of US$PPP5,599 (2009) for a 50 year old woman. The authors of the Swiss model [20] used a first-order lifetime Markov-model (individual based simulation) considering three fracture types (distal forearm, (all) vertebral and hip). They modeled all-cause mortality and excess fracture mortality for at least 5 years following hip and vertebral fractures. They considered three fracture related cost categories from a societal perspective: acute hospital care, inpatient rehabilitation and hip fracture attributable NH costs. The difference to our average fracture lifetime cost may be due to the difference in the number of modeled fracture types (3 vs. 6) and in included cost categories. We additionally included costs for fracture related informal care, professional home care and outpatient care. Furthermore we assumed that all hospitalized fractures have an influence on NH institutionalization, whereas the authors of the Swiss model assumed only hip fractures to have an impact. Additionally we modeled NH mortality, which may have had a decreasing effect on fracture attributable NH costs. Further differences in average lifetime costs may be due to different life expectancy, fracture incidence and unit costs assumptions.

A modeling study from Hiligsmann et al. [80] conducted in Belgium found discounted (3%) direct lifetime fracture costs of €10,288 (US$PPP12,102 (2009)) for women aged 55 already suffering from osteoporosis, whereas we found €7,463 (US$PPP9,329 (2009)) for a woman aged 50 years. The study’s main purpose was to evaluate the cost-effectiveness of osteoporosis screening followed by anti-osteoporotic treatment. However, the authors also reported simulated lifetime fracture costs of a 55 year old woman with osteoporosis (no intervention group). The authors used a first-order lifetime Markov model (Markov-microsimulation). They included hip, clinical vertebral, forearm and other fractures in their model. Excess mortality for hip and clinical vertebral fractures was assumed for the first and subsequent year [51]. All direct fracture costs from a health-care payer perspective were included. For hip fractures first year and long term care costs (due to a NH admission) were considered. For other fracture types only first year costs were modeled [51]. The difference in fracture lifetime cost estimates between our model and the model of Hiligsmann et al. may have various reasons. We used a parallel simulation to examine all fracture attributable NH costs with “real world” NH mortality. Hilligsmann et al. pre-calculated NH costs due to hip fractures using average life expectancy assumptions [51]. Also it seems that Belgian unit costs for hip fracture in the first year (without NH costs) are higher than our unit costs. Furthermore, we modeled excess mortality for all fracture types (except wrist) and assumed fracture excess mortality beyond the second year after a fracture, whereas Hilligsmann et al. considered excess mortality only for hip and vertebral fractures in the first two years after fracture. The difference in the cost perspective between the two modeling studies (health payer perspective vs. societal perspective) may also explain the divergence in lifetime costs.

### Strengths of the model

In comparison to other models in the field of osteoporotic fractures, our model has some strengths. Compared to a Markov cohort model our model allows calculating event probabilities dependent on the history of a modeled individual, which overcomes the classical Markov assumption of “memorylessness” [38]. Another model type which can handle patient history is the first-order Markov model (Monte Carlo simulation) where individuals are simulated separately through a classical Markov structure (health states) [38,73]. In comparison to this model type our model can handle multiple events at the same time which is not possible in a classical first-order Markov model because of the mutual exclusivity of health states [38]. Furthermore, a first-order Markov model would require a very large number of health states to reflect the same patient pathways (6 fracture types, previous fracture (yes/no), osteoporosis (yes/no), NH (yes/no)) that we considered. Applying the event based structure overcomes this “flood of health states” [30]. From an epidemiological point of view our model has strengths in terms of the number of modeled fracture types. In many models only 3 fracture types (hip, vertebra and wrist) are considered [81], whereas we modeled 6 fracture types. In addition, our model allows separating the effect of osteoporosis, previous fractures, and NH institutionalization on costs. Finally, the model validation showed that our model reflects the real world epidemiological data very well and therefore has high validity.

In terms of cost modeling, a main strength of our model is the large number of cost categories included and the way how fracture attributable long term costs are modeled. Using the parallel simulation of NH entries under real world NH mortality allowed us to estimate fracture attributable long term care costs more precisely.

### Limitations of the model

The results of model-based studies should be interpreted against the background of the model assumptions, e.g. model type, time interval, input data. In the model, we applied age-dependent fracture specific RR_fx_ only to hip fractures, but not to non-hip fractures. If the age related patterns of RR_fx_ for hip fractures are similar to other fracture types, the fracture risk for non-hip fractures in women with osteoporosis may be overestimated in older, and underestimated in younger age classes. In the model we applied osteoporosis prevalence rates and BMD data from a US population (NHANES III study). Applying these data was necessary, because no German study determined osteoporosis prevalence rates in a comparable design to the NHANES III study (in terms of osteoporosis definition based on BMD (T ≤ -2.5), sample size and study quality [41]). Furthermore, the prediction of osteoporosis risk attributions was exclusively based on BMD values measured at the femoral neck, but not at other sides, which may have led to an underestimation of osteoporosis risk attribution on other fracture sites. Fracture probabilities were mainly estimated based on the German hospital discharge statistic. In order to include fracture cases exclusively treated in an outpatient setting, fracture probabilities from the hospital discharge statistic were divided by fracture specific hospitalization probabilities. In our model these hospitalization probabilities were assumed to be age-independent. However, in a real world setting these probabilities may be age dependent and increasing with age, which would lead to an overestimation of total fracture probabilities in younger and an underestimation in older age classes. Additionally, we neglected vertebral fractures not coming to clinical attention. However, these fractures likely have a small influence on costs, but have been shown to increase the risk of subsequent fractures [82]. In terms of NH probabilities our model also underlies limitations. We assumed that only fractures treated in the hospital would increase the risk of an institutionalization in a NH. However, fractures treated in the outpatient setting may also increase the probability of an institutionalization into a NH. Hence, we may have underestimated fracture attributable long term care costs as a result. Also, we assumed that only institutionalizations within 3 months were fracture related, which could have similarly led to an underestimation of fracture attributable long term care costs.

In terms of cost data, there are further limitations. We did not consider costs due to transport, medical aids and early retirement. Cost for osteoporosis specific medication was also not considered. The main reason for neglecting medication costs was based on the aim of the study to determine fracture costs attributable to the risk factor osteoporosis, and not the cost of treating osteoporosis. A study which analyzed German insurance claims data found that the share of osteoporosis medication costs was low in comparison to other fracture attributable cost categories [83]. However, this may be due to the low osteoporosis specific treatment prevalence in Germany [83]. A further limitation is the assumption that women already residing in a NH at the time of a fracture event were assumed to not accrue fracture related long term care costs. In a real-world setting this assumption may not hold, as fracture events in a NH can go along with an increase in care needs and related costs [84]. Fracture specific long term care costs could not be presented, because all of these costs were technically attached to a single ”NH” event. Because of a lack of data we assumed age constant rehabilitation probabilities after a fracture. However, in a real world setting these probabilities may differ by age. Also we assumed that rehabilitation probabilities after a hospitalization are identical for institutionalized and community-dwelling women, which may have had an effect on rehabilitation costs. In reality institutionalized women may have lower rehabilitation probabilities. As data on outpatient resource use was only available for three fracture types (hip, vertebral and wrist), we assumed the same resource use for fractures in near bone regions (other femur, pelvis, humerus).

The estimation of expected lifetime fracture numbers and costs is highly uncertain in terms of changes in age-adjusted fracture incidence rates (secular trends). Studies from Europe showed an inconsistent picture of changes in age-adjusted fracture incidence rates, with increasing and decreasing trends reported in the last years. Potential reasons for an increase in age-adjusted hip fracture rates may be higher survival rates in very old and fragile persons, a decrease in physical activity and deficiency in vitamin D [85]. However, there are also potential reasons for a decrease in age-adjusted fracture rates, which may be due to improved medical treatment of osteoporosis (e.g. introduction of bisphosphonates), changes in habits related to fracture increasing risk factors (e.g. smoking or heavy drinking) or due to higher prevalence rates in obesity in the past years [85]. Considering the current situation of treating osteoporosis in Germany, it seems that there is room for improvements in terms of detecting and treating osteoporosis. Currently only around 18% of osteoporosis patients receive an osteoporosis specific treatment [83]. Furthermore, only 14% of fracture patients receive an osteodensitometry, which may indicate an under-detection of osteoporosis [83]. Moreover, the adherence to oral osteoporosis medications is suboptimal [86]. Improving detection and medical treatment of osteoporosis in the future could lead to a decreasing secular trend in fracture incidence rates in the future in Germany, which should also lead to a decrease in expected fracture lifetime numbers and costs. On the other hand, the predicted demographic change in Germany [22] may have an increasing effect on future age-adjusted incidence rates and therefore may increase lifetime fracture numbers and costs.

### Implications for decision makers

The results of this study show that fractures cause relevant costs in postmenopausal women, which strongly increase with age, previous fractures and osteoporosis status. The high costs in age classes over 70, in combination with demographic change, could in addition intensify the economic burden due to fractures in Germany [17]. Thus, tackling fractures in a cost-saving or cost-effective way should be an important goal for German health care decision makers.

In the future, our model can be used to perform cost-effectiveness analyses of fracture prevention interventions in Germany. Beside the described model functions, we optionally implemented an osteodensitometry module. This feature makes the modeling of different screening to treat scenarios for Germany possible. Moreover, the real-world adherence of medical anti-osteoporotic therapy can be realistically implemented in the model. As prior studies found cost-effectiveness results to be highly sensitive to medication adherence assumptions, a realistic implementation of the respective empirical evidence seems particularly important in health economic modeling of osteoporotic fractures [87].

## Conclusion

This is the first analysis modeling the epidemiology and costs of fractures for Germany. The analysis shows that 44.5% of all discounted direct fracture lifetime costs of a 50 year old woman are attributable to osteoporosis. For a 50 year old woman already suffering from osteoporosis, fracture lifetime costs are expected to be 3.3 fold the fracture costs of a healthy woman at the same age. The main cost drivers are hospital and long term care costs, causing around 70% of total fracture related lifetime costs. Beyond cost of illness, the model can be used to evaluate the cost-effectiveness of fracture prevention.

## Competing interests

The authors declare that they have no competing interests.

## Authors’ contributions

FB and HHK conceived the analysis. FB developed the simulation model, conducted the analyses and drafted the manuscript. FB and AJ prepared data as input for the model. KR, CB, HHK, FB, AJ participated in study design and implementation. HHK, AJ, KR and CB critically revised the manuscript for important intellectual content. All authors read and approved the final manuscript.

## Pre-publication history

The pre-publication history for this paper can be accessed here:

http://www.biomedcentral.com/1472-6963/14/284/prepub

## Supplementary Material

Additional file 1 **File name: Bleibler_BMCHSR_ESM.pdf (Electronic supplementary material (ESM).** Includes all used input data of the model). Click here for file
