# Supplementary material for: Expected lifetime numbers and costs of fractures in postmenopausal women with and without osteoporosis in Germany: a discrete event simulation model
Source: BMC Health Serv Res. 2014 Jun 30;14:284. doi: 10.1186/1472-6963-14-284 (PMC4118314; doi:10.1186/1472-6963-14-284)
Supplement: Additional file 1 — File name: Bleibler_BMCHSR_ESM.pdf (Electronic supplementary material (ESM). Includes all used input data of the model). [file 1472-6963-14-284-S1.pdf]

Electronic Supplementary Material

Expected lifetime numbers and costs of fractures in postmenopausal women with and without osteoporosis in Germany: A discrete event simulation model.

Florian Bleibler[1§]; Kilian Rapp[2] ; Andrea Jaensch[3]; Clemens Becker[2]; Hans-Helmut König[1]

Affiliations:

[1] Department for Health Economics and Health Service Research, Hamburg Center for Health Economics, University Medical Center Hamburg-Eppendorf, Martinistr. 52, D-20246 Hamburg, Germany

[2] Department of Clinical Gerontology, Robert-Bosch-Hospital, Auerbachstraße 110, D-70376 Stuttgart, Germany

[3] Institute of Epidemiology and Medical Biometry, Ulm University, Helmholtzstraße 22, D-89081 Ulm, Germany

§ Corresponding author

E-Mail addresses:

FB: f.bleibler@uke.de

KR: kilian.rapp@rbk.de

AJ: andrea.jaensch@uni-ulm.de

CB: clemens.becker@rbk.de

HHK: h.koenig@uke.de

Content

A. Epidemiological data 4

1. Mortality 4

a. General (female) population mortality 4

b. Relative mortality risk for women living in a nursing home compared to the female general population 4

c. Relative mortality risk for women not living in a nursing home compared to the female general population 5

d. Fracture excess mortality 6

2. Osteoporosis prevalence and incidence (calculation method) 9

3. Fracture probabilities and correction methods 9

a. Included fracture types by ICD-10 code and hospitalization probability 9

b. General population fracture probabilities by age and fracture type 10

c. Adjustment for fracture probabilities for women with and without a previous fracture 12

d. Method to determine relative fracture risk based on bone mineral density ($RR_{OST\ (i,j)}$) to calculate fracture probability for women suffering from osteoporosis. 13

e. Method for calculating fracture probabilities for women not suffering from osteoporosis ($T \geq -2.5$) 15

f. Relative fracture risks for community-dwelling women compared to the female general population by age and fracture type 16

g. Relative fracture risks for women living in a nursing home compared to the female general population by age and fracture type 16

4. Probabilities entering a nursing home 19

a. Calculation method to determine the probability of an incident admission to a long term care institution 19

b. Fracture type specific institutionalization probabilities by age 21

B. Cost data 24
1. Direct costs 24
a. Costs for acute hospital treatment per fracture case 24
b. Costs for inpatient rehabilitation treatment per fracture case 24
c. Costs for inpatient long term care 25
d. Costs for outpatient treatment per fracture case 25
e. Costs for professional home care per fracture case 25
f. Costs for informal care per fracture case 26
2. Indirect costs (Productivity costs) 26
a. Days away from work 26
b. Evaluation methods 27
C. Uncertainty assumptions for probabilistic sensitivity analysis 28
D. Validation 29
1. Internal validation 29
a. Expected vs. modeled fracture rates 29
b. Frequency of fractures per woman and fracture type 30
2. External Validation 30
a. Comparison of modeled fracture incidence rates with international epidemiological studies 30
b. Comparison of modeled fracture lifetime risk to other studies 32
E. References 33

A. Epidemiological data

1. Mortality

a. General (female) population mortality

Age	Mortality
50-54	0.0026
55-59	0.0035
60-64	0.0044
65-69	0.0066
70-74	0.0106
75-79	0.0180
80-84	0.0348
85-89	0.0692
90-94	0.1398
95+	0.2214
The female general population mortality is based on the generation life table for women born in 1959 (Variant B, Trend V2), estimated with mortality rates of the civil population. The generation life table mortalities are based on the assumption of a general decrease in mortality of women aged 60+ [1].	

b. Relative mortality risk for women living in a nursing home compared to the female general population

Age	RR	LCL95%	UCL95%
65-69	16.71	15.50	18.01
70-74	11.92	11.36	12.52
75-79	7.75	7.52	8.00
80-84	4.33	4.20	4.42
85-89	2.69	2.60	2.74
90-94	1.73	1.70	1.76
95+	1.40	1.37	1.42
Own calculation, LCL=Lower confidence limit; UCL =Upper confidence limit			

c. Relative mortality risk for women not living in a nursing home compared to the female general population

Age	RR	LCL95%	UCL95%
65-69	0.90	0.87	0.94
70-74	0.86	0.84	0.89
75-79	0.79	0.78	0.81
80-84	0.72	0.70	0.73
85-89	0.67	0.66	0.68
90-94	0.68	0.67	0.70
95+	0.70	0.68	0.72
Own calculation, LCL=Lower confidence limit; UCL =Upper confidence limit			

Data basis for section ***1b*** and ***1c*:**

Age	Community-dwelling women (n)	Death cases (n)	Women in nursing home (n)	Death cases (n)	All women (n)	Death cases (n)
65-69	722,722	5,683	4,467	650	727,189	6,333
70-74	708,256	9,006	8,978	1,580	717,234	10,586
75-79	597,116	13,202	18,556	4,025	615,672	17,227
80-84	475,933	19,349	40,427	9,918	516,360	29,267
85-89	236,079	17,369	46,051	13,630	282,130	30,999
90-94	86,101	12,281	37,383	13,554	123,483	25,835
95+	22,407	5,287	17,134	8,082	39,541	13,369
Data description: The age-dependent relative mortality risks (RR) and 95% confidence intervals (LCL/UCL95%) were calculated based on the available claims data from AOK Bavaria (own calculation). Data was pooled by age classes from 01/01/2004-31/12/2008, age was determined at the beginning of each year						

d. *Fracture excess mortality*

Data of following tables (i-vi) were taken from source [2].Only significant values were applied in the model.

i) Hip

**Age**	**Years following a fracture**	**RR**	**LCL95%**	**UCL95%**
50-59	1	21.3	3.7	121.5
50-59	2-5	3.4	1.2	3.6
50-59	6-10	2.1	1.2	3.6
60-69	1	5.1	2.1	12.3
60-69	2-5	2.5	1.7	3.6
60-69	6-10	2.3	1.7	3.1
70-79	1	3.8	3.0	5.0
70-79	2-5	1.7	1.4	2.0
70-79	6-10	1.0	1.0	1.0
80-89	1	2.0	1,6	2,6
80-89	2-5	1.0	1.0	1.0
80-89	6-10	1.0	1.0	1.0
90+	1	1.5	1.1	2.1
90+	2-5	1.0	1.0	1.0
90+	6-10	1.0	1.0	1.0

ii) Other femur (data for other fractures was used)

Age	Years following a fracture	RR	LCL95%	UCL95%
50-59	1	2.8	1.2	6.6
50-59	2-5	2	1.3	3.0
50-59	6-10	1.0	1.0	1.0
60-69	1	2.2	1.2	4.1
60-69	2-5	1.0	1.0	1.0
60-69	6-10	1.0	1.0	1.0
70-79	1	1.7	1,3	2.2
70-79	2-5	1.2	1	1.5
70-79	6-10	1.1	1	1.2
80-89	1	1.7	1.4	2.1
80-89	2-5	1.0	1.0	1.0
80-89	6-10	1.0	1.0	1.0
90+	1	1.4	1.0	1.9
90+	2-5	1.0	1.0	1.0
90+	6-10	1.0	1.0	1.0

iii) Clinical vertebral (data for vertebral was used)

Age	Years following a fracture	RR	LCL95%	UCL95%
50-59	1	3.4	1.5	7.5
50-59	2-5	2.1	1.3	3.4
50-59	6-10	1.0	1.0	1.0
60-69	1	8.3	2.4	26.6
60-69	2-5	1.0	1.0	1.0
60-69	6-10	1.6	1.3	2.0
70-79	1	3.9	2.4	6.3
70-79	2-5	1.6	1.2	2
70-79	6-10	1.0	1.0	1.0
80-89	1	1.4	1.0	2.1
80-89	2-5	1.0	1.0	1.0
80-89	6-10	1.0	1.0	1.0
90+	1	1.6	1.0	2.5
90+	2-5	1.0	1.0	1.0
90+	6-10	1.0	1.0	1.0

iv) Humerus

Age	Years following a fracture	RR	LCL95%	UCL95%
50-59	1	2.9	1.3	6.4
50-59	2-5	1.0	1.0	1.0
50-59	6-10	1.8	1.0	3.3
60-69	1	3.3	1.8	6.0
60-69	2-5	1.6	1.1	2.3
60-69	6-10	1.0	1.0	1.0
70-79	1	2	1.3	3.0
70-79	2-5	1.3	1.0	1.5
70-79	6-10	1.0	1.0	1.0
80-89	1	1.7	1.3	2.0
80-89	2-5	1.0	1.0	1.0
80-89	6-10	1.0	1.0	1.0
90+	1	1.0	1.0	1.0
90+	2-5	1.0	1.0	1.0
90+	6-10	1.0	1.0	1.0

v) Pelvis (data for other fractures was used)

Age	Years following a fracture	RR	LCL95%	UCL95%
50-59	1	2.8	1.2	6.6
50-59	2-5	2	1.3	3.0
50-59	6-10	1.0	1.0	1.0
60-69	1	2.2	1.2	4.1
60-69	2-5	1.0	1.0	1.0
60-69	6-10	1.0	1.0	1.0
70-79	1	1.7	1.3	2.2
70-79	2-5	1.2	1	1.5
70-79	6-10	1.1	1	1.2
80-89	1	1.7	1.4	2.1
80-89	2-5	1.0	1.0	1.0
80-89	6-10	1.0	1.0	1.0
90+	1	1.4	1.0	1.9
90+	2-5	1.0	1.0	1.0
90+	6-10	1.0	1.0	1.0

vi) Wrist

No excess mortality was assumed following a wrist fracture [2].

2. Osteoporosis prevalence and incidence (calculation method)

Age	Prevalence (P_t)	Incidence (Inc_t)	Method calculating incidence from prevalence
50-54	0.039	0.00269	Used formula [3]: $$Inc_t = \frac{(P_{t+1} - P_t)}{(1 - P_t)}$$ Source for prevalence P_t [4, 5] and method [3].
55-59	0.064	0.01325	
60-64	0.126	0.00755	
65-69	0.159	0.01522	
70-74	0.223	0.01982	
75-79	0.300	0.04114	
80-84	0.444	0.03561	
85-89	0.543	0.02434	
90-94	0.599	0.02772	
95+	0.654	0.03218	

Incidence was calculated based on fitted prevalence data; a linear spline fitting between the middle of each age class was applied.

3. Fracture probabilities and correction methods

a. Included fracture types by ICD-10 code and hospitalization probability

Fracture type	ICD-10 code	Hospitalisation probability	Source
Hip	S72.0-2	1.0	assumed
Other Femur	S72.3-9	1.0	assumed
Wrist	S52.5-6	0.58	[6]
Spine	S12.0-2; S12.7; S22.0-1; S32.0	0.47	See [1]
Humerus	S42.2-4	0.87	[6]
Pelvis	S32.1-8	0.75	[7]

[1] *Estimated on the assumption that 14% of all vertebral fractures are hospitalized [8] and that approx. 30% of all vertebral fractures come to clinical attention [9].*

b. General population fracture probabilities by age and fracture type

Description:

Age-dependent inpatient (acute hospital) fracture probabilities were calculated by dividing fracture cases (for each fracture type) extracted from German hospital discharge statistics (based on shown ICD-10 codes (3.a)) [10] by woman at risk (German population data from the year 2009 [11]). To estimate a total cumulative fracture incidence (see following tables 3.b. i-vi), which includes fracture cases treated in a hospital as well as in an (exclusive) outpatient setting, the hospital specific fracture probability was divided by the fracture specific hospitalization probabilities (shown above (3.a)).

i) Hip

**Age**	**Total fracture probabilities**
50-54	0.00038
55-59	0.00071
60-64	0.00104
65-69	0.00187
70-74	0.00334
75-79	0.00772
80-84	0.01605
85-89	0.02791
90-94	0.03625
95+	0.03960

ii) Other femur

**Age**	**Total fracture probabilities**
50-54	0.00010
55-59	0.00014
60-64	0.00021
65-69	0.00033
70-74	0.00054
75-79	0.00102
80-84	0.00162
85-89	0.00262
90-94	0.00324
95+	0.00382

iii) Clinical vertebral

Age	Total fracture probabilities
50-54	0.00095
55-59	0.00144
60-64	0.00192
65-69	0.00316
70-74	0.00456
75-79	0.00634
80-84	0.01132
85-89	0.01378
90-94	0.01339
95+	0.01052

iv) Humerus

Age	Total fracture probabilities
50-54	0.00085
55-59	0.00143
60-64	0.00194
65-69	0.00272
70-74	0.00360
75-79	0.00530
80-84	0.00716
85-89	0.00872
90-94	0.00861
95+	0.00795

v) Pelvis

Age	Total fracture probabilities
50-54	0.00018
55-59	0.00028
60-64	0.00038
65-69	0.00071
70-74	0.00127
75-79	0.00285
80-84	0.00544
85-89	0.00890
90-94	0.01172
95+	0.01118

vi) Wrist

Age	Total fracture probabilities
50-54	0.00221
55-59	0.00390
60-64	0.00491
65-69	0.00620
70-74	0.00684
75-79	0.00866
80-84	0.00973
85-89	0.00916
90-94	0.00740
95+	0.00530

c. *Adjustment for fracture probabilities for women with and without a previous fracture*

i) Age-independent risk ratio ($RRHip_{PreviousFx\ vs.\ NoPreviousFx}$) getting **a hip** fracture comparing women without and with a previous fracture (adjusted for bone mineral density)[12].

Age	RR	LCL95%	UCL95%
50-95+	1.56	1.23	1.98

ii) Age-independent risk ratio ($RROsteo_{PreviousFx\ vs.\ NoPreviousFx}$) getting an **osteoporotic** fracture comparing women without and with a previous fracture (adjusted for bone mineral density)[12].

Age	RR	LCL95%	UCL95%
50-95+	1.74	1.57	1.92

iii) Age independent risk ratio ($RRAny_{PreviousFx\ vs.\ NoPreviousFx}$) getting **any** fracture comparing women without and with a previous fracture (adjusted for bone mineral density)[12].

Age	RR	LCL95%	UCL95%
50-95+	1.73	1.59	1.88

iv) Prevalence of previous fractures ($Prev_{PreviousFx}$) in women by age [12].

**Age**	**%**
50	23
60	29
70	35
80	41
90	48

d. *Method to determine relative fracture risk based on bone mineral density ($RR_{OST\ (i,j)}$) to calculate fracture probability for women suffering from osteoporosis.*

Used formula [13]:

$$RR_{OST(i,j)} = \frac{\Phi((0.577 - \mu_{(j)})/\sigma + \log(RR_{fx(i,j)}))}{\Phi((0.577 - \mu_{(j)})/\sigma)}$$

Description:

Subscripts: fracture type= *i*, age=*j*

$RR_{OST\ (i,j)}$ = Relative fracture risk of a person suffering from osteoporosis (T≤-2.5) by age and fracture type, compared to the general population

Φ = Standard normal distribution function

0.577 = Osteoporosis threshold for women [13]

$\mu_{(j)}$= Mean of femoral neck BMD by age [Polynomial fit (third order), [14]) (see following table)

σ = Standard deviation of femoral neck BMD [weighted average=0.114, [14])

$RR_{fx(i,j)}$ = Relative fracture risks for 1-standard deviation decrease in BMD by fracture type (see following table).

**Age**	**Fracture type**	**RR$_{(fx(i,j))}$**	**LCL95%**	**UCL95%**	**Source**
50-54	Hip	3.68	2.61	5.19	[15]
55-59	Hip	3.35	2.51	4.47	[15]
60-64	Hip	3.07	2.42	3.89	[15]
65-69	Hip	2.89	2.39	3.50	[15]
70-74	Hip	2.78	2.39	3.23	[15]
75-79	Hip	2.58	2.30	2.90	[15]
85+	Hip	1.93	1.76	2.10	[15]
Age adjusted	Other femur	1.99	1.38	2.88	[16]
Age adjusted	Clinical vertebral	1.80	1.10	2.70	[17]
Age adjusted	Humerus	2.01	1.74	2.33	[16]
Age adjusted	Pelvis	1.82	1.46	2.27	[16]
Age adjusted	Wrist	1.40	1.40	1.60	[17]

**Age**	**Mean of femoral neck BMD [14]**
50-54	0.752
55-59	0.723
60-64	0.695
65-69	0.666
70-74	0.640
75-79	0.615
80-84	0.595
85-89	0.579
90+	0.564

e. *Method for calculating fracture probabilities for women not suffering from osteoporosis (T≥-2.5)*

Used formula [18]:

$$FxnO_{(i,j)} \approx \frac{(nPop_{(j)} * FxA_{(i,j)}) - (nPop_{(j)} * p_{(j)} * FxA_{(i,j)} * RR_{OST(i,j)})}{nPop_{(j)} - (nPop_{(j)} * p_{(j)})}$$

If

$$(nPop_{(j)} * p_{(j)} * FxA_{(i,j)} * RR_{OST(i,j)}) \geq (nPop_{(j)} * FxA_{(i,j)}),$$

100% of fractures are attributable to osteoporosis.

Description:

Subscripts:	fracture type= *i*, age=*j*
$FxnO_{(i,j)}$=	Cumulative fracture incidence for women not suffering from osteoporosis, by age and fracture type
$nPop_{(j)}$ =	Total number of women at risk in a specific age class
$FxA_{(i,j)}$=	Total cumulative fracture incidence (person with and without osteoporosis) by age and fracture type (see section 3b).
$p_{(j)}$=	Osteoporosis prevalence rate by age (see section 2).
$RR_{OST(i,j)}$ =	Relative fracture risk getting a fracture, if a woman suffers from osteoporosis by age and fracture type (see section 3b).

f. Relative fracture risks for community-dwelling women compared to the female general population by age and fracture type

i) Hip (only significant values were applied in the model)

**Age**	**RR**	**LCL95%**	**UCL95%**
65-69	0.95	0.88	1.02
70-74	0.92	0.87	0.98
75-79	0.91	0.87	0.94
80-84	0.89	0.86	0.92
85-89	0.87	0.84	0.91
90-94	0.93	0.88	0.97
95+	1.04	0.96	1.12
Own calculation			

ii) Other femur (only significant values were applied in the model)

**Age**	**RR**	**LCL95%**	**UCL95%**
65-69	0.95	0.80	1.13
70-74	0.93	0.82	1.06
75-79	0.92	0.83	1.02
80-84	0.91	0.83	1.00
85-89	0.85	0.76	0.94
90-94	0.82	0.71	0.96
95+	0.82	0.65	1.04
Own calculation			

iii) For clinical vertebral, humerus, pelvis and wrist fractures no difference were assumed/found

g. Relative fracture risks for women living in a nursing home compared to the female general population by age and fracture type

i) Hip (only significant values were applied in the model)

**Age**	**RR**	**LCL95%**	**UCL95%**
65-69	9.13	7.27	11.46
70-74	7.12	6.23	8.12
75-79	4.00	3.68	4.36
80-84	2.33	2.21	2.46
85-89	1.65	1.57	1.73
90-94	1.17	1.11	1.24
95+	0.95	0.87	1.04
Own calculation			

ii) Other femur (only significant values were applied in the model)

Age	RR	LCL95%	UCL95%
65-69	9.04	5.38	15.20
70-74	6.47	4.65	9.00
75-79	3.65	2.89	4.62
80-84	2.08	1.76	2.47
85-89	1.79	1.54	2.07
90-94	1.41	1.19	1.66
95+	1.23	0.99	1.55
Own calculation			

iii) Clinical fracture (only significant values were applied in the model)

Age	RR	LCL95%	UCL95%
65-69	2.66	1.62	4.36
70-74	2.23	1.65	3.02
75-79	1.44	1.18	1.75
80-84	1.22	1.08	1.38
85-89	0.92	0.81	1.03
90-94	0.86	0.74	0.99
95+	0.84	0.67	1.06
Own calculation			

iv) Humerus (only significant values were applied in the model)

Age	RR	LCL95%	UCL95%
65-69	3.25	2.29	4.61
70-74	2.10	1.62	2.72
75-79	1.74	1.49	2.05
80-84	1.37	1.23	1.53
85-89	1.02	0.91	1.14
90-94	1.01	0.89	1.15
95+	0.93	0.76	1.14
Own calculation			

v) Pelvis (only significant values were applied in the model)

Age	RR	LCL95%	UCL95%
65-69	1.90	0.79	4.59
70-74	4.25	3.10	5.81
75-79	2.70	2.23	3.26
80-84	1.65	1.46	1.87
85-89	1.24	1.12	1.38
90-94	1.01	0.90	1.14
95+	0.90	0.75	1.07
Own calculation			

vi) Wrist

For wrist fractures no difference was assumed/found.

Data basis for section **1e** and **1f**:

**Community-dwelling women**		**Number of fractures by type**					
**Age**	**Under consideration**	**Hip**	**Other femur**	**Clinical vertebral**	**Humerus**	**Pelvis**	**Wrist**
65-69	722,722	1,296	255	963	1,571	423	2,021
70-74	708,256	2,383	431	1,496	2,185	730	2,601
75-79	597,116	4,236	623	2,275	2,812	1,300	2,798
80-84	475,933	6,602	805	2,716	3,003	1,902	2,486
85-89	236,079	5,298	561	1,740	1,839	1,585	1,271
90-94	86,101	2,978	266	651	714	834	397
95+	22,407	1,029	100	172	189	266	107
Data description: Age dependent relative risks (RR) and 95% confidence intervals (LCL/UCL95%) were calculated based on claims data from AOK Bavaria (own calculation).<br>Data was pooled by age classes from 01/01/2004-31/12/2008, fractures were identified by admission and discharge diagnoses (ICD-10), refractures within 30 days were deleted; multiple fractures per person were possible, age was determined at the beginning of each year.							

Women living in a nursing home		Number of fractures by type					
**Age**	**Under consideration**	**Hip**	**Other femur**	**Clinical vertebral**	**Humerus**	**Pelvis**	**Wrist**
65-69	4,467	77	15	16	32	5	10
70-74	8,978	233	38	43	59	41	26
75-79	18,556	581	77	103	156	115	72
80-84	40,427	1,474	157	287	362	283	182
85-89	46,051	1,947	231	306	366	403	195
90-94	37,383	1,639	198	229	315	369	149
95+	17,134	723	115	99	128	170	49
Data description: Age dependent relative risks (RR) and 95% confidence intervals (LCL/UCL95%) were calculated based on the claims data from AOK Bavaria (own calculation). Data was pooled by age classes from 01/01/2004-31/12/2008, fractures were identified by admission and discharge diagnoses (ICD-10), refractures within 30 days were deleted; multiple fractures per person were possible, age was determined at the beginning of each year.							

4. Probabilities entering a nursing home

a. *Calculation method to determine the probability of an incident admission to a long term care institution.*

Age	Prevalence (P_t) of women in LTC by age
65-69	0.006
70-74	0.013
75-79	0.031
80-84	0.077
85-89	0.173
90-94	0.307
95+	0.475

Share (prevalence) of woman residing in long term care institution (LTC) in 2009, based on population data [11], and care statistic 2009 [19] (detailed information received per E-Mail from Federal Office of Statistics).

It was assumed that a first admission in a nursing home is not possible before the age of 65. Because there is a significant difference between the mortality of women living in a nursing home, and those that do not (see section 1.b), the following equation [3] was used to calculate the probability (incidence) of an admission to a long term care institution for any reason.

Used equation [3]: The equation was solved by applying the Newton-Raphson approach (Matlab code available from[20]) for each age class.

$$\frac{(1-P_t)P_{t+1}e^{-(\lambda_1+\lambda_2)}}{(1-P_{t+1})} = \frac{P_t e^{-\lambda_3} + (1-P_t)\left[e^{\lambda_3} - e^{-(\lambda_1+\lambda_2)}\right]\lambda_2}{(\lambda_1+\lambda_2-\lambda_3)}$$

Description:

λ_1=	Mortality rate for community-dwelling women
λ_2=	Incidence of nursing home admission (unknown) (I_{gp})
λ_3=	Mortality rate for women living in a nursing home
P_t=	Prevalence of women residing in a long term care institution in period t
P_{t+1}=	Prevalence of woman residing in a long term care institution in period t+1

**Age(j)**	**Incidence of nursing home admission gen. population (I_{gp})**	**Incidence of nursing home admission no fracture (I_{NoFx})**
67	0.0018	0.0017
72	0.0027	0.0023
77	0.0076	0.0061
82	0.0195	0.0150
87	0.0459	0.0368
92	0.0830	0.0669
97	0.1338	0.0113

The incidence of nursing home admission (I_{gp}) is valid for the general population, including those who were institutionalized due to fractures (Fx 1-6). To approximate an adjusted incidence of nursing home incidence for the population without a fracture (I_{NoFx}) we applied a method referring to an approach described by Kuntz and Weinstein [21], using the following formula:

$$I_{NoFxj} = \frac{I_{gpj} - \left(\sum_{i=1}^{6}(I_{Fxij} * Prob_{Fxij})\right)}{1 - \sum_{i=1}^{6} Prob_{Fxij}}$$

where I_{Fxij} are the age and fracture specific institutionalization probabilities and $Prob_{Fxiij}$ the fracture probabilities for women not institutionalized.

b. *Fracture type specific institutionalization probabilities by age*

Calculation method:

The age (j) and fracture type (i) specific institutionalization probabilities ($PIN_{(i,j)}$) into a long term care institution were calculated based on claims data from AOK Bavaria. All community-dwelling women aged 65 or older, hospitalized for a fracture, were considered ($W_{fx(i,j)}$). To exclude previous institutionalized women, women must live in a community-dwelling setting before the observation period (01/01/2004 and 31/01/2004). First fractures were therefore counted from the 01/01/2005 and were identified by admission and discharge diagnosis (ICD-10). Observation period was determined by 3 month after a fracture related hospitalization, death or end of follow up (30/06/2009). If a woman was institutionalized 3 month after the fracture related hospitalization, it was assumed that this institutionalization was fracture related ($IN_{fx(i,j)}$).

Therefore we used following formula to calculate the institutionalization probabilities (see tables 4.b.i-vi):

$$PIN_{(i,j)} = \frac{W_{fx(i,j)}}{IN_{fx(i,j)}}$$

(95% confidence intervals (UCL/LCL95%) were calculated based on an approximated normal distribution.)

i) Hip

Age	Wfx(i,j)	INfx(i,j))	Probability (PIN)	LCL95%	UCL95%
65-69	504	16	0.032	0.016	0.047
70-74	1,862	89	0.048	0.038	0.057
75-79	3,267	254	0.078	0.069	0.087
80-84	4,674	668	0.143	0.133	0.153
85-89	3,419	710	0.208	0.194	0.221
90-94	1,575	471	0.299	0.276	0.322
95+	421	180	0.427	0.380	0.475

ii) Other Femur

**Age**	**Wfx(i,j)**	**INfx(i,j))**	**Probability (PIN)**	**LCL95%**	**UCL95%**
65-69	92	3	0.032	-0.004	0.069
70-74	315	16	0.051	0.027	0.075
75-79	448	41	0.092	0.065	0.118
80-84	559	90	0.161	0.131	0.191
85-89	338	88	0.260	0.214	0.307
90-94	130	46	0.354	0.272	0.437
95+	48	13	0.271	0.146	0.399

iii) Clinical vertebral

**Age**	**Wfx(i,j)**	**INfx(i,j))**	**Probability (PIN)**	**LCL95%**	**UCL95%**
65-69	369	3	0.008	-0.001	0.017
70-74	1,279	28	0.022	0.014	0.030
75-79	1,885	104	0.055	0.045	0.065
80-84	2,041	212	0.104	0.091	0.117
85-89	1,240	206	0.166	0.145	0.187
90-94	362	92	0.254	0.209	0.299
95+	85	32	0.379	0.275	0.482

iv) Humerus

**Age**	**Wfx(i,j)**	**INfx(i,j))**	**Probability (PIN)**	**LCL95%**	**UCL95%**
65-69	576	7	0.012	0.003	0.021
70-74	1,725	35	0.020	0.014	0.027
75-79	2,180	84	0.039	0.030	0.047
80-84	2,184	184	0.084	0.073	0.096
85-89	1,295	201	0.155	0.136	0.175
90-94	397	124	0.313	0.267	0.358
95+	84	28	0.332	0.231	0.433

v) Pelvis

**Age**	**Wfx(i,j)**	**INfx(i,j))**	**Probability (PIN)**	**LCL95%**	**UCL95%**
65-69	149	3	0.020	-0.002	0.043
70-74	653	21	0.032	0.019	0.046
75-79	1,076	83	0.077	0.061	0.093
80-84	1,415	181	0.128	0.111	0.145
85-89	1,154	202	0.175	0.153	0.197
90-94	508	124	0.244	0.207	0.281
95+	134	48	0.359	0.278	0.440

vi) Wrist

**Age**	**Wfx(i,j)**	**INfx(i,j))**	**Probability (PIN)**	**LCL95%**	**UCL95%**
65-69	786	3	0.004	-0.0005	0.008
70-74	2,155	14	0.007	0.003	0.010
75-79	2,308	60	0.026	0.020	0.033
80-84	2,013	93	0.046	0.037	0.055
85-89	978	79	0.081	0.064	0.098
90-94	258	45	0.175	0.128	0.221
95+	56	15	0.268	0.152	0.384

B. Cost data

1. Direct costs

a. Costs for acute hospital treatment per fracture case

Fracture type	DRG based costs (operating costs)	Capital costs	Total hospital costs	Length of stay in days
Hip	6,624.00€	966.28€	7,590.28€	14.2
Other femur	6,564.90€	867.00€	7,431.90€	12.8
Clinical vertebral	4,337.66€	758.88€	5,096.50€	11.2
Humerus	4,358.06€	622.20€	4,980.26€	9.2
Pelvis	3,358.36€	683.40€	4,041.76€	10.1
Wrist	2,716.80€	293.08€	3,009.90€	4.3

Following state weighted base rate 2,887€ for the year 2009[22] was used to calculate DRG costs. Main source to calculate DRG costs (weighted relative weighs and lengths of stay) was the German DRG Browser V2010 (data year 2009)[23]. Capital costs per case were calculated by multiplying capital costs per day (68€) with the fracture type specific length of stay. Capital costs per day were calculated based on the method described in Krauth et. al. [24] using following assumptions: Investment cost for a hospital bed in 2001: 251,000€ [24] Relation between building and assets: 2/3 to 1/3 [24]; Operating life expectancy for building: 50 years [24];Operating life expectancy for assets: 10 years [24];Interest: 0.03[24]; Inflation correction to 2009 for building [25] and assets [26];Bed occupancy rate: 0.775[27]

b. Costs for inpatient rehabilitation treatment per fracture case

Fracture type	Rehabilitation cost per case €	Length of stay in days	Reha probability after a hospital stay
Hip	2,187€	21.38	0.310
Other femur	2,187€	21.38	0.310
Clinical vertebral	2,092€	20.45	0.053
Humerus	2,337€	21.87	0.079
Pelvis	2,177€	21.28	0.12
Wrist	2,337€	22.84	0.009

Inpatient rehabilitation unit costs (per day 102.3€) for the year 2009 were calculated based on statistics from the statutory pension insurance [28], dividing total costs for rehabilitation by all rehabilitation days (excluding rehabilitation costs and days for mental disorders) in 2009 .Rehabilitation probabilities were approximated based on a disease statistic from a large statutory health insurance company [29], dividing fracture related hospital cases by the rehabilitation cases per year. Length of stay in a rehabilitation institution was taken from the same source [29].

c. *Costs for inpatient long term care*

	**Operating costs per day**	**Capital costs per day**	**Cost per year**
	57.00€	13.53€	25,758.70€
Operating costs per day is a weighted average, based on the level of care (I-III) distribution of female long term care residents from source [19], Capital costs per day are taken from source [30]. Daily cost for board and accommodation were excluded.			

d. *Costs for outpatient treatment per fracture case*

**Fracture type**	**Outpatient treatment after hospitalization**[1]	**Exclusive outpatient treatment**[1]
Hip	963.40€	n.A.
Other femur	963.40€	n.A.
Clinical vertebral	1,227.40€	1.613.90€
Humerus	783.50€	834.80€
Pelvis	963.40€	963.40€
Wrist	783.50€	834.80€
Included costs components: Outpatient surgeon, physician and physiotherapy visits as well as analgesic therapy. Number of physician, surgeon and physiotherapy visits as well amount of medication (analgesics) per fracture was taken from a German cost-effectiveness study [31]. Resource use (visits) was valued with German unit prices from Krauth et al. [24] (inflated to the year 2009 with the German consumer price index [32]). Medication was valued using the Red List 2009 [33], using a conservative approach (lowest price). Because no resource use data for humerus, pelvis and other femur fractures was available. It was assumed that the costs for humerus and wrist as well as the costs for pelvis, other femur and hip fractures are equivalent.		

e. *Costs for professional home care per fracture case*

**Fracture type**	**Cost per case**	**Amount of used hours per case**
Hip	2,174€	58
Other femur	2,174€	58
Clinical vertebral	2,212€	59
Humerus	937.3€	25
Pelvis	2,174€	58
Wrist	524.9€	14
An hourly cost rate of 37.5€ was assumed [34]. The authors of the original source calculated 27 different cost per hour variants from 27.9€-50.4€. 37.5€ per hour corresponds to the mean of all variants [34]. The amount of used hours per case was taken from an Austrian source [35]. It was assumed that the consumed hours are equivalent for other femur, hip and pelvis.		

f. Costs for informal care per fracture case

**Fracture type**	**Cost per case**	**Amount of used hours per case**
Hip	2,361.2€	130
Other femur	2,361.2€	130
Clinical vertebral	2,016.1€	111
Humerus	2,960.6€	163
Pelvis	2,361.2€	130
Wrist	581.2€	32
In order to value informal care we used the market cost method [36]. An hourly wage rate of 14.24€ for a person working in the area of care for elderly and disabled persons were applied. (Q88.1 [37],detailed information received per E-Mail from Federal Office of Statistics) The hourly wage rate was corrected with a factor of 1.275 correcting for employee contributions to social security and other employee labour costs [38, 39].(Corrected and applied hourly wage rate: 18.16€). The amount of hours per case was taken from an Austrian source [35]. It was assumed that the consumed hours are equivalent for other femur, hip and pelvis.		

2. Indirect costs (Productivity costs)

a. Days away from work

**Fracture type**	**Average days away from work**	
Hip	50.97	
Other femur	50.97	
Clinical vertebral	29.09	
Humerus	44.94	
Pelvis	53.92	
Wrist	43.36	
Source [29], days for rehabilitation are not included, therefore see section B.1.b.		

b. Evaluation methods

Age	Employment rate[1]	Yearly average gross salary[2]
50-55	0.746	24,575€
55-60	0.631	24,575€
60-65	0.303	24,575€
65-75	0.026	24,575€
>75	0	24,575€

The human capital [39] and friction cost approach [40] was applied. The yearly gross salary was adjusted with a factor of 1.275 correcting for employee contributions to social security and other employee labour costs [38, 39]. A yearly wage increase of around 2% was assumed ([41],[39]). For women aged 75 years or over an employment rate of zero was assumed. In terms of friction costs we assumed a friction period of 72 days [42].If the total absence period was shorter as the friction period 80% of costs were applied [40]. Productivity costs were estimated by taking the difference in gained productivity between women at average risk getting osteoporosis and those never get osteoporosis.

[1]Employment rates from Germans and foreign residents [43].

[2] Yearly average gross salary is based on full and all part time employments [37]

C. Uncertainty assumptions for probabilistic sensitivity analysis

**Epidemiological data**		
Category	*Section*	*Distributional assumption*
Mortality (except fracture excess mortality)	A.1.a-c	N.A. (full and large sample)
Fracture excess mortality	A.1.d	Log-Normal
Osteoporosis prevalence	A.2	Uniform +/- 30%
Hospital probabilities	A.3.a	Beta
General fracture probabilities	A.3.b	N.A. (full sample)
Relative fracture risks for 1-standard deviation decrease in BMD by fracture type	A.3.d	Log-Normal
Relative fracture risks for community-dwelling woman compared to the female general population by age and fracture type	A.3.f	Log-Normal
Relative fracture risks for women living in a nursing home compared to the female general population by age and fracture type	A.3.g	Log-Normal
Age dependent risk ratio (RR) getting any/hip/osteoporotic fracture comparing women without and with a previous fracture	A.3.c	Log Normal
Prevalence of previous fracture	A.3.c	Uniform +/-30%
Prevalence of women in LTC by age (Basis for transformation in incidence)	A.4.a	N.A. (full sample)
Fracture type specific institutionalization probabilities by age	A.4.b	Beta
**Cost data**		
Category	*Section*	*Distributional assumption*
DRG based cost	B.1.a	N.A. (full sample)
Capital costs	B.1.a	None
Reha cost per case	B.1.b	N.A. (large sample)
Reha probability	B.1.b	Beta
Cost for inpatient long term care	B.1.c	N.A. (full sample)
Cost for outpatient treatment	B.1.d	Gamma
Professional home care /hourly cost rate	B.1.e	Gamma
Hours informal/professional care	B.1.e-d	None
Indirect costs	B.2	None

D. Validation

1. Internal validation

We performed an internal validation to verify that the model was correctly implemented. Therefore we compared our expected fracture rates (input data) with the modeled fracture rates (D.1.a). Also we present the number of fractures per woman for each fracture type and all fractures types together (D.1.b).

a. Expected vs. modeled fracture rates

	**Hip**		**Other femur**		**Cl. Vertebral**	
Age	expected	modeled	expected	modeled	expected	modeled
50-54	0.00038	0.00034	0.00010	0.00010	0.00095	0.00092
55-59	0.00071	0.00065	0.00014	0.00015	0.00144	0.00145
60-64	0.00104	0.00108	0.00021	0.00020	0.00192	0.00201
65-69	0.00187	0.00189	0.00033	0.00038	0.00316	0.00303
70-74	0.00334	0.00344	0.00054	0.00060	0.00456	0.00494
75-79	0.00772	0.00763	0.00102	0.00114	0.00634	0.00786
80-84	0.01605	0.01563	0.00162	0.00162	0.01132	0.01202
85-90	0.02791	0.02684	0.00262	0.00268	0.01378	0.01426
90-95	0.03625	0.03552	0.00324	0.00328	0.01339	0.01460
95+	0.03960	0.03998	0.00382	0.00434	0.01052	0.01127

	**Humerus**		**Pelvis**		**Wrist**	
Age	expected	modeled	expected	modeled	expected	modeled
50-54	0.00085	0.00079	0.00018	0.00017	0.00221	0.00215
55-59	0.00143	0.00132	0.00028	0.00026	0.00390	0.00385
60-64	0.00194	0.00201	0.00038	0.00042	0.00491	0.00484
65-69	0.00272	0.00277	0.00071	0.00070	0.00620	0.00606
70-74	0.00360	0.00385	0.00127	0.00140	0.00684	0.00697
75-79	0.00530	0.00555	0.00285	0.00322	0.00866	0.00875
80-84	0.00716	0.00762	0.00544	0.00602	0.00973	0.01003
85-90	0.00872	0.00901	0.00890	0.00983	0.00916	0.00956
90-95	0.00861	0.00923	0.01172	0.01225	0.00740	0.00796
95+	0.00795	0.00843	0.01118	0.01204	0.00530	0.00563

b. Frequency of fractures per woman and fracture type

**Fractures per women**	**Hip**	**Other Femur**	**Clinical Vertebral**	**Humerus**	**Pelvis**	**Wrist**	**All**
0	77.63%	96.80%	82.38%	86.88%	90.45%	80.47%	48.03%
1	17.57%	3.10%	14.99%	11.66%	8.70%	16.52%	25.13%
2	3.92%	0.09%	2.32%	1.31%	0.79%	2.66%	14.19%
3	0.75%	0.00%	0.27%	0.14%	0.06%	0.33%	7.22%
4	0.12%	0.00%	0.03%	0.01%	0.00%	0.03%	3.25%
5	0.02%	0.00%	0.00%	0.00%	0.00%	0.00%	1.36%
6	0.00%	0.00%	0.00%	0.00%	0.00%	0.00%	0.54%
Basis: 200,000 simulated women							

2. External Validation

a. Comparison of modeled fracture incidence rates with international epidemiological studies

Country	Author	Year	Add. Information
Sweden	Kanis et al [44]	1987-1993	
UK	Singer et al [45]	1992-1993	Only metaphyseal fractures
USA	Ettinger et al [46]	2006	
Germany (modeled)	Bleibler	2009	

i) Hip fracture incidence rate for different countries

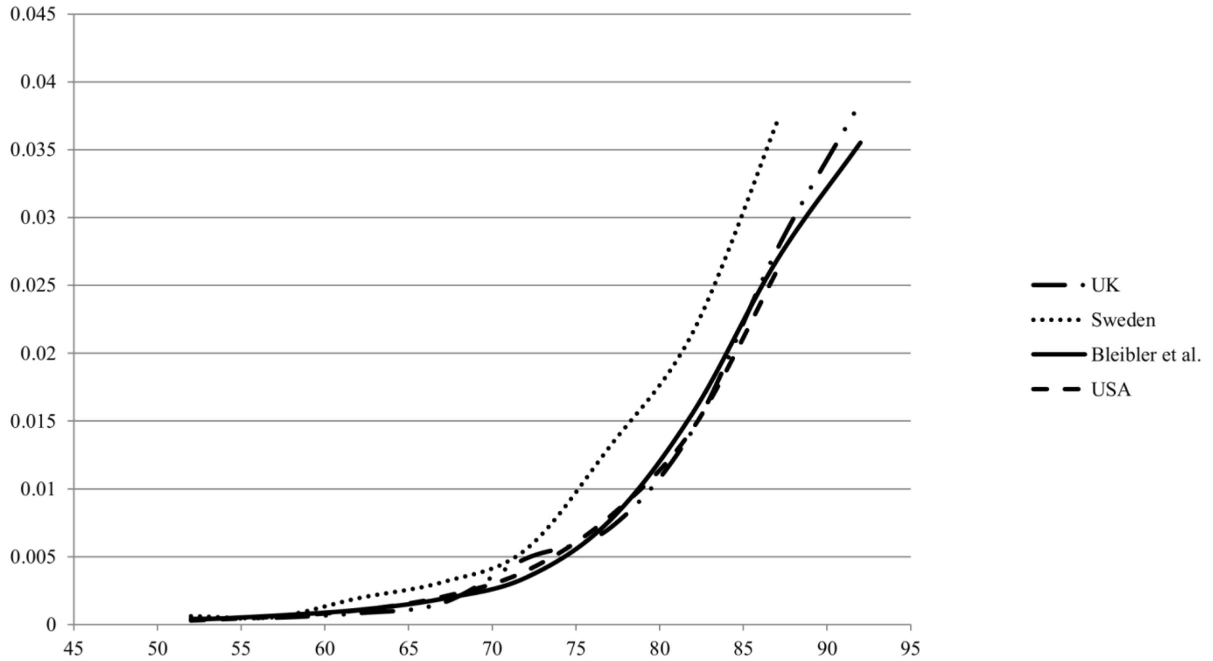

ii) Clinical vertebral fracture incidence rate for different countries

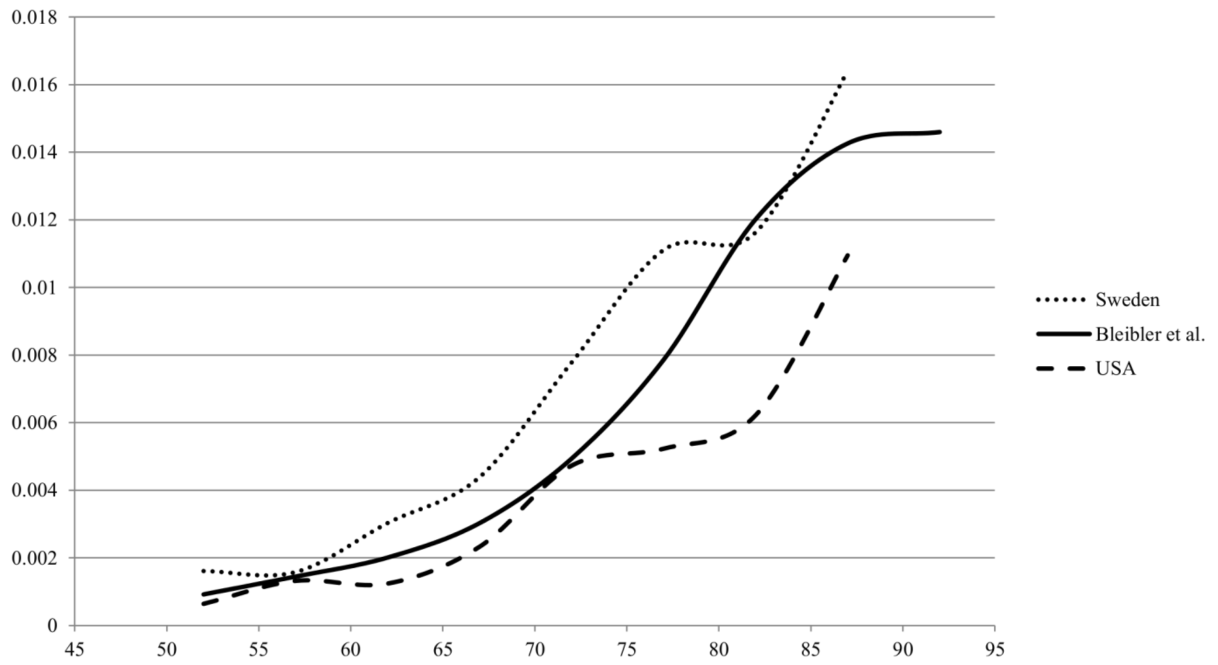

iii) Wrist fracture incidence rate for different countries

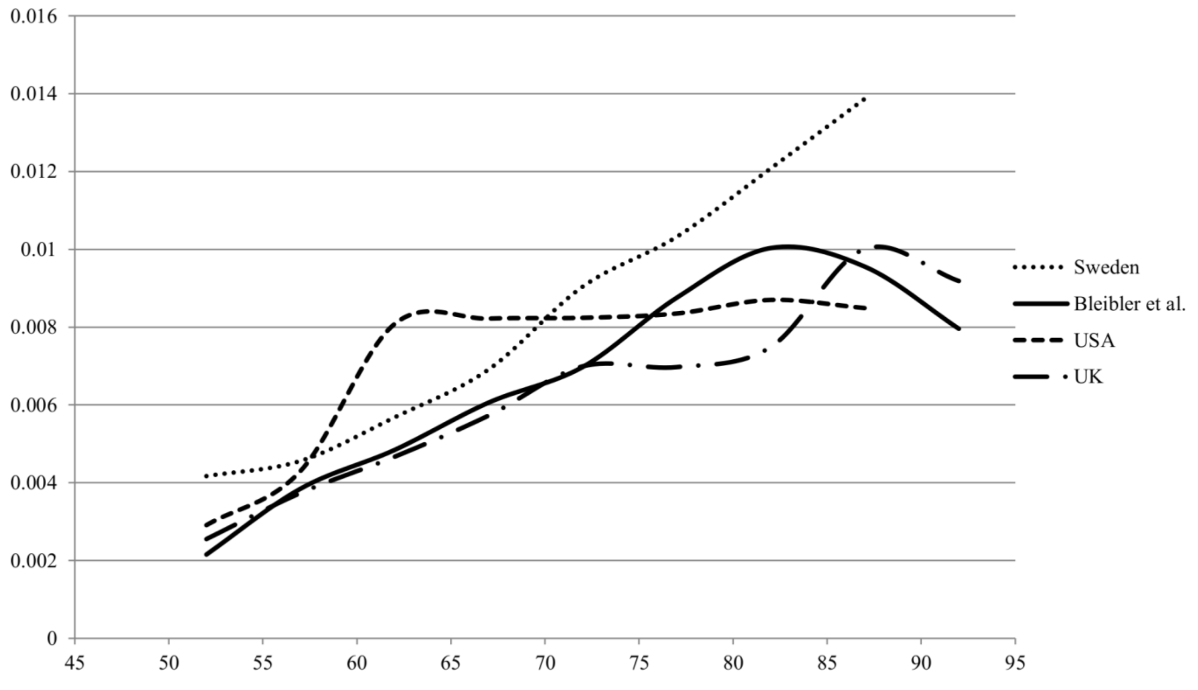

iv) Humerus fracture incidence rate for different countries

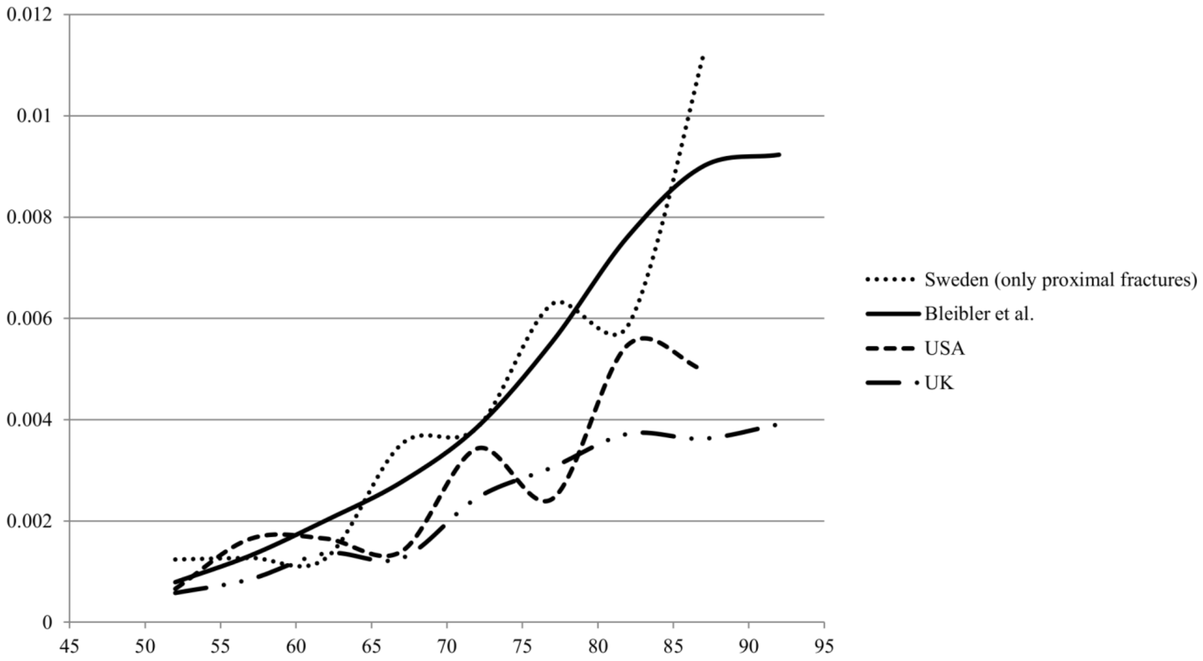

b. Comparison of modeled fracture lifetime risk to other studies

**Fracture Lifetime risk of a 50 year old woman ***								
**Country**	**Typ**	**Hip**	**Other Femur**	**Clinical Vertebral**	**Hum.**	**Pelvis**	**Wrist**	**All**
**GER [C]**	**M**	**19.8%**	**3.1%**	**16.2%**	**12.4%**	**9.1%**	**18.0%**	**35.8%**
**BE [47]**	M	29.0%	x	x	x	X	x	44.3%
**AUS [48]**	M	17.0%	1.5%	9.6%	x	3.8%	13.3%	42.1%
**CH [49]**	M	20.9%	x	42.6% (all)	x	x	13.8%	x
**USA**[50]**	M	24.0%	x	28.0% (all)	x	x	17.0%	x
**SWE [44]**	E	22.9%	x	15.1%	12.9%	x	20.8%	46.4%
**USA [51]**	E	17.5%	x	15.6%	x	x	16.0%	39.7%
[C]=Present study; M=Modeling study; E=Epidemiological study , ** 65 year old women, *First ever lifetime risk								

E. References

1. Statistisches Bundesamt [Federal Statistical Office]: **Generationensterbetafeln für Deutschland:Modellrechnung für die Geburtenjahrgange 1871-2004 [Generation lifetables for Germany: Model calculation for the birth cohorts 1871-2004].** Wiesbaden (Germany); 2006.

2. Morin S, Lix LM, Azimaee M, Metge C, Caetano P, Leslie WD: **Mortality rates after incident non-traumatic fractures in older men and women.** *Osteoporos Int* 2011, **22:**2439-2448.

3. Podgor MJ, Leske MC: **Estimating incidence from age-specific prevalence for irreversible diseases with differential mortality.** *Stat Med* 1986, **5:**573-578.

4. Jerusel N: **Analyse und Prognose der Krankheitslast Osteoporose-attributabler proximaler Femurfrakturen in Deutschland [Analysis and Prognosis of the health burden of osteoporosis attributable proximal femur fractures in Germany].** *PhD thesis.* University Leipzig (Germany), Health Economics Research Unit 2009.

5. Wildner M: **Osteoporose [Osteoporosis].** *Dtsch med Wochenschr* 2001**:**126:A1170-A1172.

6. Einsiedel T, Becker C, Stengel D, Schmelz A, Kramer M, Daxle M, Lechner F, Kinzl L, Gebhard F: **[Do injuries of the upper extremity in geriatric patients end up in helplessness? A prospective study for the outcome of distal radius and proximal humerus fractures in individuals over 65].** *Z Gerontol Geriatr* 2006, **39:**451-461.

7. Boufous S, Finch C, Close J, Day L, Lord S: **Hospital admissions following presentations to emergency departments for a fracture in older people.** *Inj Prev* 2007, **13:**211-214.

8. Finnern HW, Sykes DP: **The hospital cost of vertebral fractures in the EU: estimates using national datasets.** *Osteoporos Int* 2003, **14:**429-436.

9. Cooper C, O'neill T, Silman A: **The epidemiology of vertebral fractures.** *Bone* 1993, **14:**89-97.

10. Statistisches Bundesamt [Federal Statistical Office]: **Tiefgegliederte Diagnosedaten der Krankenhauspatentinnen und -patienten 2009 [Deep stratified diagnosis-data from female and male inpatients 2009].** Wiesbaden (Germany); 2011.

11. Statistisches Bundesamt [Federal Statistical Office]: **Bevölkerung Deutschland bis 2060:Ergebnisse der 12. koordinierten Bevölkerungsvorausbrechnung [Population in Germany up to the year 2060:Results of the 12th coordinated projection of population].** Wiesbaden (Germany); 2009.

12. Kanis JA, Johnell O, De Laet C, Johansson H, Oden A, Delmas P, Eisman J, Fujiwara S, Garnero P, Kroger H, et al: **A meta-analysis of previous fracture and subsequent fracture risk.** *Bone* 2004, **35:**375-382.

13. Kanis JA, Johnell O, Oden A, Jönsson B, De Laet C, Dawson A: **Risk of hip fracture according to the World Health Organization criteria for osteopenia and osteoporosis.** *Bone* 2000, **27:**585-590.

14. Looker AC, Wahner HW, Dunn WL, Calvo MS, Harris TB, Heyse SP, Johnston CC, Jr., Lindsay R: **Updated data on proximal femur bone mineral levels of US adults.** *Osteoporos Int* 1998, **8:**468-489.

15. Johnell O, Kanis JA, Oden A, Johansson H, De Laet C, Delmas P, Eisman JA, Fujiwara S, Kroger H, Mellstrom D, et al: **Predictive value of BMD for hip and other fractures.** *J Bone Miner Res* 2005, **20:**1185-1194.

16. Stone KL, Seeley DG, Lui LY, Cauley JA, Ensrud K, Browner WS, Nevitt MC, Cummings SR, Osteoporotic Fractures Research G: **BMD at multiple sites and risk of fracture of multiple types: long-term results from the Study of Osteoporotic Fractures.** *J Bone Miner Res* 2003, **18:**1947-1954.

17. Marshall D, Johnell O, Wedel H: **Meta-analysis of how well measures of bone mineral density predict occurrence of osteoporotic fractures.** *BMJ* 1996, **312:**1254-1259.

18. Bleibler F, Konnopka A, Benzinger P, Rapp K, König HH: **The health burden and costs of incident fractures attributable to osteoporosis from 2010 to 2050 in Germany--a demographic simulation model.** *Osteoporos Int* 2013, **24:**835-847.

19. Statistisches Bundesamt [Federal Statistical Office]: **Pflegestatistik 2009 Pflege im Rahmen der Pflegeversicherung Deutschlandergebnisse [Statistics on care 2009.Care in the context of the mandaratory care insurance. Results from Germany].** Wiesbaden(Germany); 2011.

20. **Solution of non-linear equations** [http://ocw.usu.edu/Civil_and_Environmental_Engineering/Numerical_Methods_in_Civil_Engineering/NonLinearEquationsMatlab.pdf]

21. Kuntz KM, Weinstein MC: **Modelling in economic evaluation.** *Economic evaluation in health care: merging theory with practice* 2001**:**141-171.

22. Deutsche Krankenhaus Gesellschaft [German Hospital Association]: **Landesbasisfallwerte der Bundesländer [Baserates of the German Federal States]**. Berlin (Germany); 2010. [http://www.dkgev.de/media/file/7827.LBFW_2005_2010_Stand_160610.pdf]

23. Institut für das Entgeltsystem im Krankenhaus [Institute for the Hospital Remuneration System]: **G-DRG V2010 Browser (2009 § 21 KHEntgG)**. Siegburg (Germany); 2011.[http://www.g-drg.de/cms/Archiv/Systemjahr_2011_bzw._Datenjahr_2009#sm15]

24. Krauth C, Hessel F, Hansmeier T, Wasem J, Seitz R, Schweikert B: **[Empirical standard costs for health economic evaluation in Germany -- a proposal by the working group methods in health economic evaluation].** *Gesundheitswesen* 2005, **67:**736-746.

25. Statistisches Bundesamt [Federal Statistical Office]: **Preisindizies für die Bauwirtschaft Tabelle 61261-0001 (Price indices for construction Table 61261-0001).** Statistisches Bundesamt; 2013.[https://www-genesis.destatis.de/genesis/online/data;jsessionid=53A6983D00246CB81D27F08661290897.tomcat_GO_1_2?operation=abruftabelleBearbeiten&levelindex=2&levelid=1361178107502&auswahloperation=abruftabelleAuspraegungAuswaehlen&auswahlverzeichnis=ordnungsstruktur&auswahlziel=werteabruf&selectionname=61261-0001&auswahltext=%23SBAUAR1-BPNG2%23SBAUAR4-BAULEISTBW%23Z-01.01.2010%2C01.01.2009%2C01.01.2008%2C01.01.2007%2C01.01.2006%2C01.01.2005%2C01.01.2004%2C01.01.2003%2C01.01.2002%2C01.01.2001%2C01.01.2000&werteabruf=Werteabruf]

26. Statistisches Bundesamt [Federal Statistical Office]: **Preisindizies für gewerbliche Produkte (Maschinen) Tabell 61241-0001 [Price indices for commercial products (machines) Table 61241-0001].** Statistisches Bundesamt; 2013. [https://www-genesis.destatis.de/genesis/online/data;jsessionid=53A6983D00246CB81D27F08661290897.tomcat_GO_1_2?operation=abruftabelleBearbeiten&levelindex=2&levelid=1361176124541&auswahloperation=abruftabelleAuspraegungAuswaehlen&auswahlverzeichnis=ordnungsstruktur&auswahlziel=werteabruf&selectionname=61241-0001&auswahltext=%23SGP09Y2-GP09-28%23Z-01.01.2011%2C01.01.2010%2C01.01.2009%2C01.01.2008%2C01.01.2007%2C01.01.2006%2C01.01.2005%2C01.01.2004%2C01.01.2003%2C01.01.2002%2C01.01.2001&nummer=5&variable=2&name=GP09Y2]

27. Statistisches Bundesamt [Federal Statistical Office]: **Grundaten der Krankenhäuser [Basis data of German hospitals].** Wiesbaden (Germany); 2011.

28. Deutsche Rentenversicherung [German statutory pension insurance]: **Rehabilitation 2009.** Berlin (Germany); 2010.

29. AOK Bundesverband [AOK Federal Association]: **Krankheitsartenstatistik 2008 [Disease statistic 2008]** Berlin (Germany); 2009.

30. Bundesministerium für Gesundheit [Federal Ministry of Health]: **Drucksache 17/8332 - Fünfter Bericht über die Entwicklung der Pflegeversicherung und den Stand der pflegerischen Versorgung in der Bundesrepublik Deutschland [Printed matter 17/8332- Fifth report on the development of the mandaratory care incurance and the current status of nursing care in the Federal Republic of Germany].** Berlin (Germany).

31. Kreck S, Klaus J, Leidl R, von Tirpitz C, Konnopka A, Matschinger H, König HH: **Cost effectiveness of ibandronate for the prevention of fractures in inflammatory bowel disease-related osteoporosis: cost-utility analysis using a Markov model.** *Pharmacoeconomics* 2008, **26:**311-328.

32. Statistisches Bundesamt [Federal Statistical Office]: **Preise - Verbraucherpreisindizes für Deutschland (Lange Reihen ab 1948) [Prices - consumer price indices for Germany (Long series from 1948)].** Wiesbaden (Germany); 2013.

33. Rote Liste Service GmbH: *Rote Liste 2009 [Red List 2009]*. Frankfurt am Main (Germany): Rote Liste Service GmbH; 2009.

34. Rothgang H: **Vergütung von Leistungen der Häuslichen Krankenpflege nach § 37 SGB V [Reimbursement of home care services in accordance with § 37 SGB V]** In *Oral presentation at: 4Bayrischer Tag der ambulanten Pflege des bpa [Forth Bavarian day of home care by bpa]* Munich (Germany); 2011. [http://www.sfb597.uni-bremen.de/homepages/rothgang/downloads/110328_Rothgang_Verguetung_von_Leistungen_der_Haeuslichen_Krankenpflege%20_bpa.pdf]

35. Dimai HP, Redlich K, Schneider H, Siebert U, Viernstein H, Mahlich J: **Direkte und indirekte Kosten von osteoporotisch bedingten Frakturen in Osterreich [Direct and indirect costs of fractures due to osteoporosis in Austria].** *Gesundheitswesen* 2012, **74:**e90-98.

36. van den Berg B, Brouwer WB, Koopmanschap MA: **Economic valuation of informal care. An overview of methods and applications.** *Eur J Health Econ* 2004, **5:**36-45.

37. Statistisches Bundesamt [Federal Statistical Office]: **Verdienst und Arbeitskosten 2009 [Earning and labour costs 2009].**Wiesbaden (Germany); 2010.

38. **Social security and other labour costs paid by employer % of total labour costs (10 employees or more)** [http://epp.eurostat.ec.europa.eu/tgm/refreshTableAction.do;jsessionid=9ea7d07d30da009798d90cda41d8b89fadddd0393cc1.e34MbxeSaxaSc40LbNiMbxeNaxuRe0?tab=table&plugin=1&pcode=tps00114&language=en]

39. Rice DP, Cooper BS: **The economic value of human life.** *Am J Public Health Nations Health* 1967, **57:**1954-1966.

40. Koopmanschap MA, Rutten FF, van Ineveld BM, van Roijen L: **The friction cost method for measuring indirect costs of disease.** *J Health Econ* 1995, **14:**171-189.

41. Eurostat: **Annual net earnings** [http://appsso.eurostat.ec.europa.eu/nui/show.do?dataset=earn_nt_net&lang=eng]

42. Heckmann M, Kettner A, Rebien K, Vogler-Ludwig K: **Unternehmensbefragung im IV. Quartal 2009: Stellenbesetzung in Zeiten der Krise(IAB Kurzbericht 23/2010) [The German Job Vacancy Survey in the IVth quarter of 2009 : Staffing in times of crisis (IAB short report 23/2010)].** vol. 23/2010. Nürnberg (Germany); 2010.

43. Statistisches Bundesamt [Federal Statistical Office]: **Mikrozensus Bevölkerung und Erwerbstätigkeit Stand und Entwicklung der Erwertstätigkeit Deutschland 2009 [Microcensus Population and Employment Status quo and developement of employment Germany 2009].** vol. 1. Wiesbaden (Germany); 2010.

44. Kanis J, Johnell O, Oden A, Sernbo I, Redlund-Johnell I, Dawson A, De Laet C, Jonsson B: **Long-term risk of osteoporotic fracture in Malmö.** *Osteoporosis international* 2000, **11:**669-674.

45. Singer B, McLauchlan G, Robinson C, Christie J: **Epidemiology of fractures in 15 000 adults the influence of age and gender.** *Journal of Bone & Joint Surgery, British Volume* 1998, **80:**243-248.

46. Ettinger B, Black D, Dawson-Hughes B, Pressman A, Melton III L: **Updated fracture incidence rates for the US version of FRAX®.** *Osteoporosis international* 2010, **21:**25-33.

47. Hiligsmann M, Bruyère O, Ethgen O, Gathon H-J, Reginster J-Y: **Lifetime absolute risk of hip and other osteoporotic fracture in Belgian women.** *Bone* 2008, **43:**991-994.

48. Doherty DA, Sanders KM, Kotowicz MA, Prince RL: **Lifetime and five-year age-specific risks of first and subsequent osteoporotic fractures in postmenopausal women.** *Osteoporos Int* 2001, **12:**16-23.

49. Schwenkglenks M, Lippuner K, Häuselmann HJ, Szucs TD: **A model of osteoporosis impact in Switzerland 2000–2020.** *Osteoporosis international* 2005, **16:**659-671.

50. Nayak S, Roberts MS, Greenspan SL: **Cost-effectiveness of different screening strategies for osteoporosis in postmenopausal women.** *Annals of internal medicine* 2011, **155:**751-761.

51. Melton LJ, Chrischilles EA, Cooper C, Lane AW, Riggs BL: **How many women have osteoporosis?** *Journal of bone and mineral research* 2005, **20:**886-892.
